# An emerging plume head interacting with the Hawaiian plume tail

**DOI:** 10.1016/j.xinn.2023.100404

**Published:** 2023-02-22

**Authors:** Lipeng Zhang, Zebin Cao, Robert E. Zartman, Congying Li, Saijun Sun, Lijun Liu, Weidong Sun

**Affiliations:** 1 Center of Deep Sea Research, Institute of Oceanology, Chinese Academy of Sciences, Qingdao 266071, China; 2 Deep-Sea Multidisciplinary Research Center, Laoshan Laboratory, Qingdao 266237, China; 3 Department of Geology, University of Illinois at Urbana-Champaign, Urbana, IL 61820, USA; 4 Laboratory for Marine Geology, Laoshan Laboratory, Qingdao 266237, China; 5 University of the Chinese Academy of Sciences, Beijing 100049, China

## Abstract

The Hawaiian-Emperor seamount chain has shown two subparallel geographical and geochemical volcanic trends, Loa and Kea, since ∼5 Ma, for which numerous models have been proposed that usually involve a single mantle plume sampling different compositional sources of the deep or shallow mantle. However, both the dramatically increased eruption rate of the Hawaiian hotspot since ∼5 Ma and the nearly simultaneous southward bending of the Hawaiian chain remain unexplained. Here, we propose a plume-plume interaction model where the compositionally depleted Kea trend represents the original Hawaiian plume tail and the relatively enriched Loa trend represents an emerging plume head southeast of the Hawaiian plume tail. Geodynamic modeling further suggests that the interaction between the existing Hawaiian plume tail and the emerging Loa plume head is responsible for the southward bending of the Hawaiian chain. We show that the arrival of the new plume head also dramatically increases the eruption rate along the hotspot track. We suggest that this double-plume scenario may also represent an important mechanism for the formation of other hotspot tracks in the Pacific plate, likely reflecting a dynamic reorganization of the lowermost mantle.

## Public summary

• Hawaiian hotspot track displays two subparallel isotopic trends since 5 Ma.
• This is coeval with increased eruption rate and southward bending of the track.
• These data support a new plume head emerging next to the old Hawaiian plume.
• Our double-plume model may represent a new mechanism for hotspot evolution.

## Graphical abstract

**Figure:**
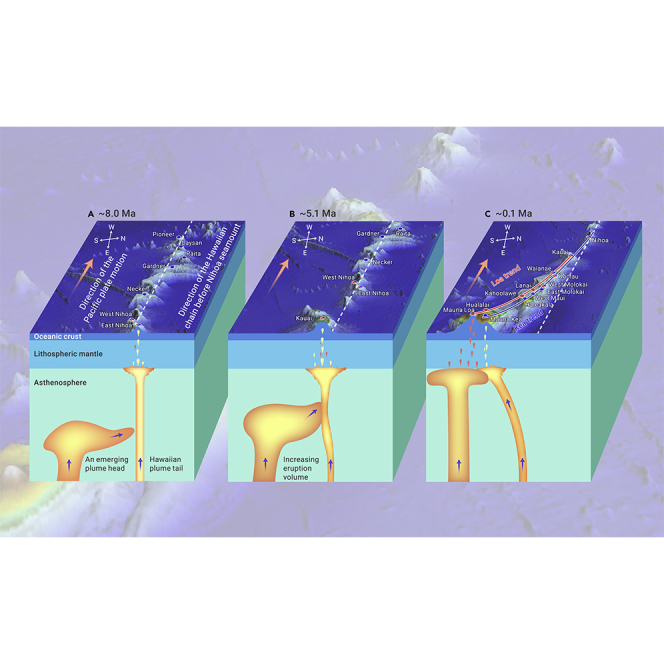

## Introduction

**Figure 1:**
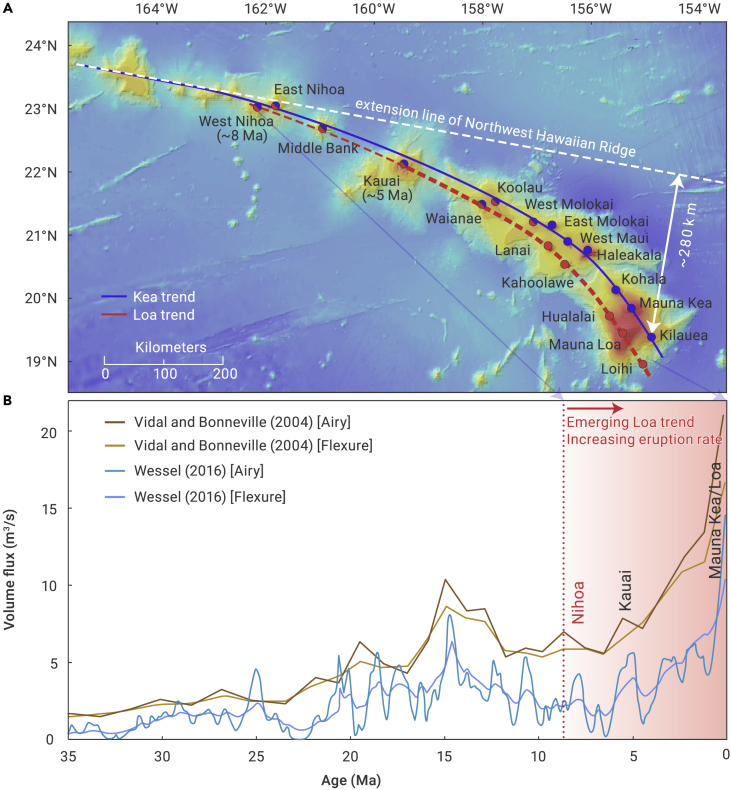
Map showing double chains of the Hawaiian hotspot The bending of the Hawaiian chains started at ∼8 Ma. The Kea trend (north) is the original Hawaiian chain, whereas the Loa trend (south) is newly emerged as indicated by isotopic characteristics (see Figure 2). The Nihoa seamount (∼8 Ma) is the first volcano that has samples with geochemical signatures of the Loa trend, and it became clearly discernable at Kauai (∼5 Ma). Correspondingly, the volume and magma flux of the Hawaiian chain has increased dramatically since ∼5 Ma (Figure 1B). Volume flux is estimated based on the airy and flexural compensation models.^26^^,^^27^ The latest eruption rate is ∼21 m^3^/s, i.e., 0.66 km^3^/year. Interestingly, double chains are also reported for the Samoa and the Marquesas volcano chains (Figure S1). Geochronology data from previous literature of the seamounts are compiled in the supplemental information.

**Figure 2:**
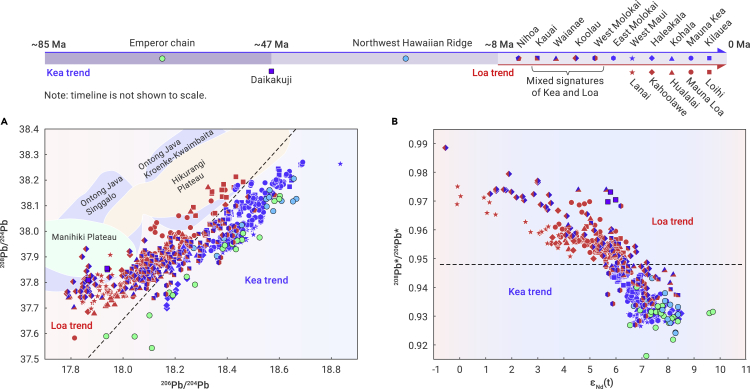
Isotope diagrams for shield-stage lavas of Hawaiian-Emperor volcano chains The Hawaiian-Emperor chain is divided into three sections: the Emperor chain, the Northwest Hawaiian Ridge, and the seamounts form Nihoa to the present position. (A) ^208^Pb/^204^Pb versus ^206^Pb/^204^Pb. The Hawaiian chain is divided into two distinctive trends, the Loa trend and the Kea trend, by the dashed line. Note that all samples from the Emperor chain and the Northwest Hawaiian Ridge except the Daikakuji seamount fall on the Kea trend, suggesting that the Kea trend was the original Hawaii chain, whereas the Loa trend is newly emerging. Nihoa, Kauai, Waianae, Koolau, and West Molokai have large ranges of isotopic compositions and fall on both trends, i.e., mixtures of materials from two trends at shallow depths. (B) A diagram of ^208^Pb∗/^206^Pb∗ versus ε_Nd_(t) where ^208^Pb∗/^206^Pb∗ represents the time-integrated ^232^Th/^238^U ratio since the formation of the Earth and is defined as ^208^Pb∗/^206^Pb∗ = (^208^Pb/^204^Pb–29.475)/(^206^Pb/^204^Pb–9.306).^28^ Samples from the Loa trend all have ^208^Pb∗/^206^Pb∗ higher than 0.948, with lower ε_Nd_(t), representing an enriched mantle source. In contrast, samples from the Kea trend all have ^208^Pb∗/^206^Pb∗ lower than 0.948, with higher ε_Nd_(t), comparable to those of the depleted mantle. The sources of geochemical data of these seamounts are provided in the supplemental information.

The conventional plume hypothesis predicts that a single chain of volcanos with age progression should form as a plate passes over a relatively stationary mantle plume.^1^^,^^2^ The Hawaiian-Emperor chain, however, does not strictly follow this prediction. The volcanos of the Hawaiian chain progressively bifurcated into two subparallel geographical and geochemical trends since ∼5 Ma: the Loa and the Kea trends (Figure 1). These two trends are of significant compositional differences in major and trace elements and are best illustrated by the contrasting Pb and Nd isotopes. Overall, the Loa trend has lower ε_Nd_ and higher ^208^Pb∗/^206^Pb∗ compared with the Kea trend and with mid-ocean ridge basalts (Figures 1 and 2).^3^^,^^4^^,^^5^^,^^6^^,^^7^^,^^8^^,^^9^^,^^10^^,^^11^^,^^12^^,^^13^^,^^14^^,^^15^^,^^16^ The two isotopically distinct trends of the Hawaiian hotspot were interpreted previously using a variety of models.^5^^,^^6^^,^^7^^,^^8^^,^^13^^,^^16^^,^^17^^,^^18^^,^^19^^,^^20^^,^^21^^,^^22^ For example, it has been suggested that the Hawaiian plume is concentrically zoned in chemical and isotopic compositions, with the Loa trend sampling the center and the Kea trend sampling the margins.^17^^,^^23^^,^^24^ Based on high-precision Pb isotopes, it is proposed that the two trends have very little compositional overlap such that they originated from bilateral, non-concentric plume zones.^5^^,^^15^^,^^25^ The geochemical differences between the Kea and Loa trends were also explained by two distinct sources at the core-mantle boundary.^6^^,^^8^^,^^11^^,^^13^^,^^14^^,^^16^ Another study proposed that the chemical variation of the Hawaiian mantle plume was controlled by its thermal structure.^7^ A recent study attributed the double trends to a recent azimuthal change in the motion of the Pacific plate such that the Loa trend sampled the shallow portions of the plume dominated by a pyroxenite melt zone, while the Kea trend sampled the deep peridotite melt zone at the center of the Hawaii plume.^18^ However, the drift direction of the Pacific plate has changed multiple times in the geological history, but these changes do not always correlate with subparallel hotspot chains showing distinct geochemical characteristics within the Pacific plate. In brief, none of the published models could explain the fact that the bifurcated geographical and geochemical trends did not appear until ∼5 Ma, a phenomenon not only observed in Hawaii (Figures 1 and 2) but also in other hotspot tracks (Figure S1).

## Results and discussion

### The synchronous occurrence of geochemical bifurcation, bending of the seamount chain, and rapidly increasing eruption rates

According to geochemical data, the Loa trend began to appear in the Nihoa seamount (∼8 Ma)^16^ and became clearly discernable in the Kauai seamount (∼5 Ma).^5^^,^^6^^,^^8^^,^^13^^,^^14^^,^^16^^,^^29^ To the west of the Nihoa seamount, the Hawaiian chain, including the entire Emperor chain, is classified as belonging to the Kea trend (Figures 1 and 2). Therefore, the Kea trend represents the original Hawaiian plume tail (Figure 1) that has lasted for more than 80 Myr, whereas the Loa trend should be a new one (<8 Myr)^8^^,^^13^^,^^30^ with enriched isotope compositions, which are typical for plume heads in the Pacific plate, such as Ontong Java, Manihiki, and Hikurangi (Figure 2).^31^^,^^32^^,^^33^^,^^34^^,^^35^^,^^36^^,^^37^ Some researchers found that some samples from the Daikakuji seamount have enriched geochemical signatures.^13^^,^^14^^,^^38^^,^^39^ This is at least partially due to alterations.^39^ Furthermore, their major and trace element and isotope compositions show obvious differences from those of the Loa trend.^38^^,^^39^ Especially for the most representative Pb isotope of the Loa trend, the Daikakuji samples do not lie exactly on the main field of the Loa trend (Figure 2). It erupted near the Hess Rise and thus may have been influenced by underplated enriched magmas from the southern Hess Rise, which has high radiogenic Pb isotopes and low Nd isotopes.^31^ Previous studies have also attributed these isolated isotope characteristics of Daikakuji to the presence of a small mantle heterogeneity,^13^^,^^14^ which is essentially different from the seamounts (<8 Ma) of the Loa trend.

Remarkably, the occurrence of bifurcated geochemical characteristics in the Hawaiian magmas in <8 Ma is synchronous with the abrupt bending of the hotspot chain (Figure 1). Starting roughly at ∼8–5 Ma, the Hawaiian chain bends to the south by ∼45° from its original trajectory (defined by the Northwest Hawaiian Ridge), with a total offset of ∼280 km by now (Figure 1). This was attributed to the recent azimuthal change in Pacific plate motion.^18^ However, no such bent has been observed in other seamount chains on the Pacific plate during the same period (Figure S1), which precludes the changing plate motion as the cause.

According to the classical plume hypothesis, a mantle plume is characterized by a large head that usually forms a large igneous province at the surface, followed by a long, thin tail that corresponds to a volcano chain.^1^^,^^2^^,^^40^^,^^41^^,^^42^^,^^43^^,^^44^^,^^45^^,^^46^ The Hawaiian-Emperor volcano chain is generally attributed to the tail of the Hawaiian plume. However, the eruption rate of the Hawaiian plume has increased dramatically in the last few million years (Figure 1).^27^^,^^47^^,^^48^ The main island of Hawaii, with an estimated volume of ∼0.21 million cubic kilometers,^48^ is much larger than volcanos within most other hotspot chains. The average eruption rate of the Hawaiian plume was mostly ∼0.017 km^3^/year between 47.5 and 5 Ma. This suddenly increased to ∼0.05 km^3^/year at ∼5 Ma at Kauai,^48^ contemporaneous with the commencement of the southward bending (Figure 1). The total eruption rate of the Hawaiian plume sharply increased again to ∼0.66 km^3^/year (i.e., ∼21 m^3^/s) at the latest stage,^26^^,^^27^ ∼40 times higher than the average eruption rate between 47.5 and 5 Ma and even comparable to that of a plume head. For example, the Deccan Trap,^49^ a well-studied large igneous province erupted from an underlying plume head, has a total volume of ∼0.7 million cubic kilometers,^1^ erupted within one million years, which corresponds to an average eruption rate of ∼0.7 km^3^/year. It is followed by a thin island chain to the south, formed by the eruption of the Reunion mantle plume.^50^^,^^51^ The distinctive eruption behavior of the Hawaiian plume seems to contradict the mantle plume hypothesis, which predicts that the eruption rate of the plume tail should be significantly lower than that of the plume head.^1^^,^^13^^,^^52^ It is important to note that the rapidly increasing eruption rate of the Hawaiian plume in the last few million years is due to the growth of the Loa trend, which became clearly discernable at ∼5 Ma (Figures 1 and 2).

**Figure 3:**
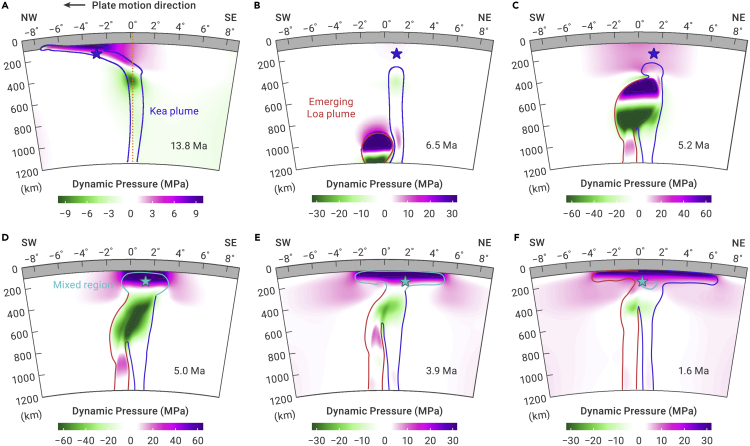
Temporal evolution of the double-plume system (A) The established Hawaiian plume at 13.8 Ma, prior to the commencement of the Loa plume. This cross-section is along the plate motion direction. The gray area and blue line represent the overlying oceanic lithosphere and the plume conduit, respectively. Within the plume conduit, dynamic pressure ranges from −9 to 6 MPa, with positive values right beneath the LAB and negative values in the deeper plume conduit. The dark blue stars denote spatial centers of the Kea plume system at 200 km depth. (B–F) Snapshots of the double-plume system at 6.5, 5.2, 5.0, 3.9, and 1.6 Ma, respectively. The light blue stars denote spatial centers of the double-plume system at 200 km depth. All cross-sections are perpendicular to the plate motion direction along the orange line in (A).

### An emerging plume head interacting with the original Hawaiian plume

All these observations can be better explained by the interaction of an emerging plume head with the waning Hawaiian plume tail. We name this new plume the Loa plume. The root of the Loa plume was located to the southeast of that of the Hawaiian plume, and the subsequent southward migration of the hotspot chain, the increasing magma eruption rates, and the progressive separation of the two geochemical trends reflect the dynamic interaction and competition of the two plumes as they strive to dominate the source of surface volcanism. Nihoa, which erupted ∼8 Ma, provides the first compositional signal for a different mantle source (Figures 1 and 2), which we interpret as representing the arrival of the Loa plume head to the mantle melt zone.

To better understand this plume-plume interaction, we designed a high-resolution 3D geodynamic model using the code CitcomS.^53^ The model starts with an established Hawaiian plume conduit that has a diameter of 100 km and a growing Loa plume head whose conduit is twice as wide and 250 km further south, both rooted at 1,500 km depth. More model details can be found in the supplemental information. We track the movement of the Loa plume head as it approaches the surface and interacts with the Hawaiian plume.

**Figure 4:**
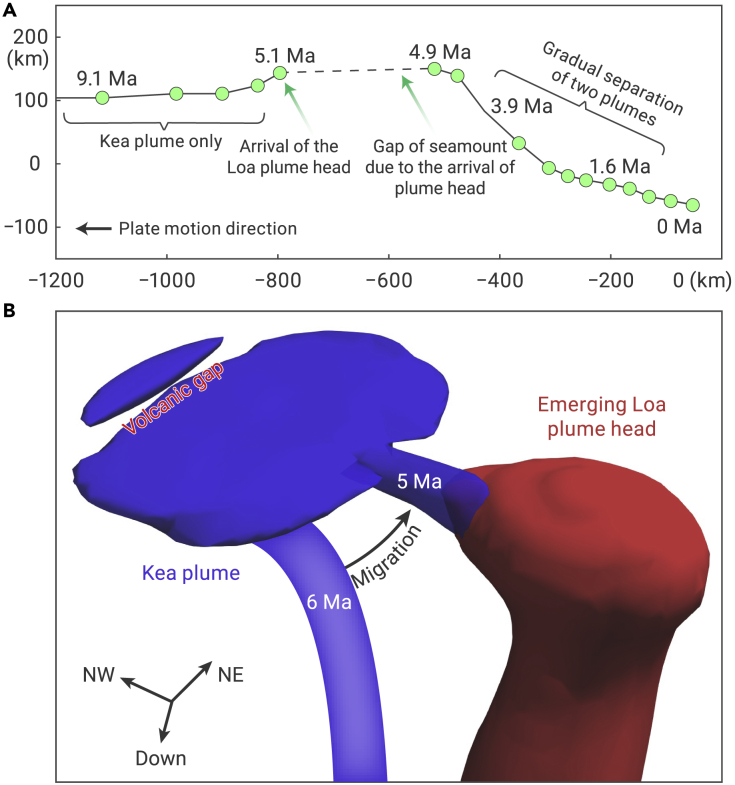
Predicted migration history of plume system centers and temporal evolution of the double-plume system in 3D view (A) Current locations of plume system centers identified at different time. The horizontal axis shows the distance to the current plume system center along the plate motion direction, where negative distance means that old centers are located west to the current center. The vertical axis shows the distance to current plume center in the direction perpendicular to the plate motion, where positive distance means that old centers are located north to the current center. Numbers next to the dots indicates the volcano ages. (B) The emerging Loa plume head causes the Kea plume conduit to migrate southeastward during 6–5 Ma, where the resulting ∼200 km volcanic gap is comparable to the observed surface magmatic hiatus.

As a plume ascends, the upper half of the plume head and the region above experience positive dynamic pressure due to upward compression. The lower plume head and the region further below experience negative dynamic pressure due to extension. Because the nearby Hawaiian plume produces a conduit of negative dynamic pressure throughout the mantle (Figure 3A), the rising Loa plume head is initially attracted by the Hawaiian plume conduit following the lateral pressure gradient (Figures 3B and 3C). Prior to the arrival of the Loa plume, the Hawaiian conduit is entrained northwestward following the Pacific plate motion (Figures 3A and S3). Due to the larger buoyancy than that of the Hawaiian tail, the Loa plume head rises faster and more vertically. In addition, the temporally reduced mantle viscosity due to increased temperature helps alleviate plate entrainment so that the location of the Loa plume head around the lithosphere-asthenosphere boundary (LAB) jumped eastward by ∼200 km, leading to a clear gap of plume flux along the direction of plate motion. Consequently, this creates a magmatic hiatus along the Hawaiian conduit. In observation, there was a ∼200 km wide gap in the Hawaiian chain without seamounts to the west of Kauai when the bending started (Figure 1). This is consistent with the modeled plume-plume interaction (Figure 4).

Because the strong lateral pressure gradient diverts the Loa plume head toward the Hawaiian conduit, the Loa plume initially reaches the LAB at a similar location as the original Hawaiian conduit, which is aligned with the earlier Hawaiian chain downstream of the Pacific motion direction (Figure 3). Initial mixing of the two plumes implies that magmas with Loa characteristics should start to appear along the original Hawaiian chain, as is observed starting in Nihoa, and the islands/seamounts from Nihoa to West Molokai display both Loa and Kea geochemical signatures before the distinct Loa trend has fully emerged (Figure 1). Their geochemical compositions vary dramatically, plotted on both the Loa and Kea trends (Figure 2), which clearly indicate “mixing” of the Kea and Loa magmas, and also with the depleted mantle.^8^^,^^17^ The highest ^208^Pb∗/^206^Pb∗ and lowest ε_Nd_ values among all Hawaiian basalts are seen in Koolau, implying that these Koolau samples are close to the Loa end member (Figure 2). However, the trace elements in inclusions of Koolau basalts are highly varied and show geochemical characteristics of both Kea and Loa.^7^

After the plume head settles down in the asthenosphere, its upward motion slows down. Accordingly, the positive dynamic pressure within the plume head decreases rapidly (Figures 3D and 3E). Consequently, the reduced lateral pressure difference from that of the Hawaiian conduit (the Kea trend) means that the Loa plume is driven mostly by its own buoyancy and can rise more vertically. This results in a gradual shift of its lateral location away from the older Hawaiian chain and toward the south (Figures 3E and 3F). Meanwhile, the eruption rate of the emerging plume head increases dramatically. Given that the Loa plume is broader and hotter than the Hawaiian plume tail conduit, the latter will eventually lean toward the former, whose more-negative dynamic pressure exerts a stronger suction force (Figures 3E and 3F). Finally, the two plume conduits stay close to, but remain separated from, each other, which could explain the two distinct compositional trends in the Hawaiian hotspot. Since ∼5 Ma, the center of the plume system has experienced a continuous southward migration over ∼175 km at 200 km depth (Figure 4), which is comparable to the southward migration of the surface hotspot (Figure 1).

This plume-plume interaction model we proposed is further supported by geophysical results. Global seismic tomography shows that there are two distinct anomalies underneath Hawaii.^54^ The S-wave imaging also shows two low-velocity regions at depths of ∼600 km. However, at depths of 900 and 1,200 km, the anomalous region to the northwest of the Hawaiian main island becomes vague, while that to the southeast is still clearly shown.^55^ These results may imply that the Hawaiian plume tail is located to the northwest of the main island and is dying, whereas the young Loa plume is located to the southeast and is growing over time.

### Implications on plume dynamics and resulting surface manifestation

According to the conventional plume hypothesis, the mantle source of plumes is largely stationary,^2^^,^^44^ such that the island/seamount chain formed from volcanic eruptions within a moving plate. Paleomagnetic data showed that several Emperor seamounts older than 50 Ma erupted at paleolatitude far north of the current position, which has been interpreted due to southward migration of the plume in response to a change in the locus of upwelling mantle flow.^56^^,^^57^^,^^58^^,^^59^ A recent study shows that this could be instead due to the interaction between the plume and the northward moving ridge between the Pacific and Izanagi plates.^60^

Here, we show that the surface expression of hotspots may also reflect a plume-plume interaction. Most previous models for Cenozoic variations in the Hawaiian chain involve a single mantle plume originating from either the ambient Pacific mantle or the large low shear-wave velocity province (LLSVP).^5^^,^^6^^,^^8^^,^^13^^,^^23^^,^^24^^,^^25^ Of particular notice is the model by Harrison et al.,^13^ who proposed that the once distant Hawaiian mantle plume sampling the ambient Pacific lowermost mantle moved toward and eventually interacted with the spatially fixed Pacific LLSVP, when the plume started to entrain the LLSVP material to form the Loa trend. This model satisfies multiple constraints but is inconsistent with geodynamic simulations showing that plumes tend to form above a temporally evolving LLSVP.^61^^,^^62^^,^^63^ In addition, this model cannot explain the southward bending of the Hawaiian chain nor the recent increase in eruption rate.

In our model, both plumes could have originated from the top of the LLSVP. Their different isotopic signatures could be due to multiple reasons. One possibility is that their respective source regions have different compositions. A second possibility is that both plumes sample the same composition (i.e., the LLSVP), but their abilities to entrain the LLSVP material differ: the waning plume tail entrains less material than the newly formed plume head due to their different buoyancy and thus suction force applied to the underlying plume source. A third explanation is that both plumes entrain similar amounts of LLSVP material but the new plume head, due to its higher temperature and lack of prior melting, could express a stronger compositional signature.

Dynamically, the recent appearance of the Loa plume next to the older one may reflect a regional perturbation or reorganization of the convective regime within the lowermost mantle. According to recent geodynamic studies,^62^^,^^63^ the LLSVP’s configuration evolves over time due to push of the surrounding mantle and subducting slabs. We suggest that the formation of the new plume may be a result of this temporal variation. Furthermore, this recent lowermost mantle adjustment may also explain the two subparallel isotopic trends in Samoa and the Marquesas Islands that have similar ages to the Loa trend (Figure S1). We speculate that these hotspot tracks together reflect the same mechanism, i.e., an emerging new plume next to the old one, in response to a regional reorganization of the Pacific lower mantle. We propose that future work is needed to better understand the causes and consequences of this late-Cenozoic change in mantle dynamic regime.

Mantle plume heads are often associated with large igneous provinces,^1^^,^^41^^,^^45^ which can cause major global climate changes.^64^^,^^65^^,^^66^^,^^67^ It has been proposed that the Deccan large igneous province in India was responsible for the mass extinction at the end of the Cretaceous.^64^ It could also have contributed to the extremely hot climate in the early Cenozoic by adding CO_2_ to the atmosphere. The eruption rate in Hawaii today is rapidly increasing, which we propose is due to invigoration from an emerging plume head. Considering the potentially major influences this increasing volcanic activity may have on the habitability of planet Earth, such a scenario is worthy of attention. However, if CO_2_ in mantle plumes comes mainly from carbonated domains deep inside the mantle’s transition zone,^68^ the volatiles in the mantle domains underneath Hawaii may have already been undergoing extraction for more than 80 Myr. The emerging plume head would then likely be much more depleted in CO_2_ and other volatile components compared with the Deccan plume head.

## Materials and methods

Materials and methods related to this work are available in the supplemental information.

## Lead contact website

http://ir.qdio.ac.cn/profile/weidongsun.

## Supplemental information

Document S1. Figures S1–S3, Tables S1 and S2, and supplemental information

Document S2. Article plus supplemental information

## References

[1] Campbell I.H. (2005). Large igneous provinces and the mantle plume hypothesis. Elements.

[2] Morgan W.J. (1971). Convection plumes in lower mantle. Nature.

[3] Tatsumoto M. (1978). Isotopic composition of lead in oceanic basalt and its implication to mantle evolution. Earth Planet Sci. Lett..

[4] Hauri E.H. (1996). Major-element variability in the Hawaiian mantle plume. Nature.

[5] Abouchami W., Hofmann A.W., Galer S.J.G. (2005). Lead isotopes reveal bilateral asymmetry and vertical continuity in the Hawaiian mantle plume. Nature.

[6] Huang S., Hall P.S., Jackson M.G. (2011). Geochemical zoning of volcanic chains associated with Pacific hotspots. Nat. Geosci..

[7] Ren Z.-Y., Ingle S., Takahashi E. (2005). The chemical structure of the Hawaiian mantle plume. Nature.

[8] Weis D., Garcia M.O., Rhodes J.M. (2011). Role of the deep mantle in generating the compositional asymmetry of the Hawaiian mantle plume. Nat. Geosci..

[9] Jackson M.G., Weis D., Huang S. (2012). Major element variations in Hawaiian shield lavas: source features and perspectives from global ocean island basalt (OIB) systematics. Geochem. Geophys. Geosyst..

[10] Frey F.A., Huang S., Xu G., Jochum K.P. (2016). The geochemical components that distinguish Loa- and Kea-trend Hawaiian shield lavas. Geochem. Cosmochim. Acta.

[11] Harrison L.N., Weis D., Garcia M.O. (2020). The multiple depleted mantle components in the Hawaiian-Emperor chain. Chem. Geol..

[12] Pietruszka A.J., Norman M.D., Garcia M.O. (2013). Chemical heterogeneity in the Hawaiian mantle plume from the alteration and dehydration of recycled oceanic crust. Earth Planet Sci. Lett..

[13] Harrison L.N., Weis D., Garcia M.O. (2017). The link between Hawaiian mantle plume composition, magmatic flux, and deep mantle geodynamics. Earth Planet Sci. Lett..

[14] Harrison L.N., Weis D. (2018). The size and emergence of geochemical heterogeneities in the Hawaiian mantle plume constrained by Sr-Nd-Hf isotopic variation over similar to 47 million years. Geochem. Geophys. Geosyst..

[15] Xu G., Huang S., Frey F.A. (2014). The distribution of geochemical heterogeneities in the source of Hawaiian shield lavas as revealed by a transect across the strike of the Loa and Kea spatial trends: east Molokai to West Molokai to Penguin Bank. Geochem. Cosmochim. Acta.

[16] Williamson N.M.B., Weis D., Scoates J.S. (2019). Tracking the geochemical transition between the kea-dominated northwest Hawaiian ridge and the bilateral loa-kea trends of the Hawaiian islands. Geochem. Geophys. Geosyst..

[17] Sobolev A.V., Hofmann A.W., Sobolev S.V., Nikogosian I.K. (2005). An olivine-free mantle source of Hawaiian shield basalts. Nature.

[18] Jones T.D., Davies D.R., Campbell I.H. (2017). The concurrent emergence and causes of double volcanic hotspot tracks on the Pacific plate. Nature.

[19] Hofmann A.W., Farnetani C.G. (2013). Two views of Hawaiian plume structure. Geochem. Geophys. Geosyst..

[20] Farnetani C.G., Hofmann A.W. (2010). Dynamics and internal structure of the Hawaiian plume. Earth Planet Sci. Lett..

[21] Ballmer M.D., Ito G., van Hunen J., Tackley P.J. (2011). Spatial and temporal variability in Hawaiian hotspot volcanism induced by small-scale convection. Nat. Geosci..

[22] Xu G., Frey F.A., Clague D.A. (2007). Geochemical characteristics of West Molokai shield- and postshield-stage lavas: constraints on Hawaiian plume models. Geochem. Geophys. Geosyst..

[23] Lassiter J.C., DePaolo D.J., Tatsumoto M. (1996). Isotopic evolution of mauna Kea volcano: results from the initial phase of the Hawaii scientific drilling project. J. Geophys. Res..

[24] Bryce J.G., DePaolo D.J., Lassiter J.C. (2005). Geochemical structure of the Hawaiian plume: Sr, Nd, and Os isotopes in the 2.8 km HSDP-2 section of Mauna Kea volcano. Geochem. Geophys. Geosyst..

[25] Huang S., Abouchami W., Blichert-Toft J. (2009). Ancient carbonate sedimentary signature in the Hawaiian plume: evidence from Mahukona volcano.

[26] Wessel P. (2016). Regional–residual separation of bathymetry and revised estimates of Hawaii plume flux. Geophys. J. Int..

[27] Vidal V., Bonneville A. (2004). Variations of the Hawaiian hot spot activity revealed by variations in the magma production rate. J. Geophys. Res..

[28] Galer S.J.G., Onions R.K. (1985). Residence time of thorium, uranium and lead in the mantle with implications for mantle convection. Nature.

[29] Garcia M.O., Swinnard L., Weis D. (2010). Petrology, geochemistry and geochronology of Kaua‘i lavas over 4·5 Myr: implications for the origin of rejuvenated volcanism and the evolution of the Hawaiian plume. J. Petrol..

[30] Weis D., Harrison L.N., McMillan R., Williamson N.M.B. (2020). Fine-Scale structure of earth's deep mantle resolved through statistical analysis of Hawaiian basalt geochemistry. Geochem. Geophys. Geosyst..

[31] Tejada M.L.G., Geldmacher J., Hauff F. (2016). Geochemistry and age of shatsky, hess, and ojin rise seamounts: implications for a connection between the shatsky and hess rises. Geochem. Cosmochim. Acta.

[32] Tejada M.L.G., Mahoney J.J., Neal C.R. (2002). Basement geochemistry and geochronology of central malaita, Solomon Islands, with implications for the origin and evolution of the Ontong Java plateau. J. Petrol..

[33] Tejada M.L.G., Mahoney J.J., Castillo P.R. (2004). Pin-pricking the elephant: evidence on the origin of the Ontong Java Plateau from Pb-Sr-Hf-Nd isotopic characteristics of ODP leg 192 basalts. Origin and Evolution of the Ontong Java Plateau.

[34] Mahoney J.J., Spencer K.J. (1991). Isotopic evidence for the origin of the Manihiki and Ontong Java oceanic plateaus. Earth Planet Sci. Lett..

[35] Ingle S., Mahoney J.J., Sato H. (2007). Depleted mantle wedge and sediment fingerprint in unusual basalts from the Manihiki Plateau, central Pacific Ocean. Geology.

[36] Hoernle K., Hauff F., van den Bogaard P. (2010). Age and geochemistry of volcanic rocks from the Hikurangi and Manihiki oceanic Plateaus. Geochem. Cosmochim. Acta.

[37] Timm C., Hoernle K., Werner R. (2011). Age and geochemistry of the oceanic Manihiki Plateau, SW Pacific: new evidence for a plume origin. Earth Planet Sci. Lett..

[38] Garcia M.O., Smith J.R., Tree J.P. (2015).

[39] Tree J.P. (2016).

[40] Campbell I.H., Griffiths R.W. (1990). Implications of mantle plume structure for the evolution of flood basalts. Earth Planet Sci. Lett..

[41] Coffin M.F., Eldholm O. (1994). Large igneous provinces-crustal structure, dimensions, and external consequences. Rev. Geophys..

[42] Koppers A.A.P., Becker T.W., Jackson M.G. (2021). Mantle plumes and their role in Earth processes. Nat. Rev. Earth Environ..

[43] Whitehead J.A., Luther D.S. (1975). Dynamics of laboratory diapir and plume models. J. Geophys. Res..

[44] French S.W., Romanowicz B. (2015). Broad plumes rooted at the base of the Earth'smantle beneath major hotspots. Nature.

[45] Richards M.A., Duncan R.A., Courtillot V.E. (1989). Flood basalts and hot-spot tracks: plume heads and tails. Science.

[46] Farnetani C.G., Samuel H. (2005). Beyond the thermal plume paradigm. Geophys. Res. Lett..

[47] Wessel P., Müller R.D. (2016). Ridge-spotting: a new test for Pacific absolute plate motion models. Geochem. Geophys. Geosyst..

[48] Robinson J.E., Eakins B.W. (2006). Calculated volumes of individual shield volcanoes at the young end of the Hawaiian Ridge. J. Volcanol. Geoth. Res..

[49] Self S., Widdowson M., Thordarson T., Jay A.E. (2006). Volatile fluxes during flood basalt eruptions and potential effects on the global environment: a Deccan perspective. Earth Planet Sci. Lett..

[50] Bredow E., Steinberger B., Gassmöller R., Dannberg J. (2017). How plume-ridge interaction shapes the crustal thickness pattern of the Réunion hotspot track. Geochem. Geophys. Geosyst..

[51] Peters B.J., Day J.M.D. (2017). A geochemical link between plume head and tail volcanism. Geochem. Perspect. Lett..

[52] Griffiths R.W., Campbell I.H. (1991). Interaction of mantle plume heads with the earths surface and onset of small-scale convection. J. Geophys. Res..

[53] Zhong S., McNamara A., Tan E. (2008). A benchmark study on mantle convection in a 3-D spherical shell using CitcomS. Geochem. Geophys. Geosyst..

[54] Montelli R., Nolet G., Dahlen F.A. (2004). Finite-frequency tomography reveals a variety of plumes in the mantle. Science.

[55] Wolfe C.J., Solomon S.C., Laske G. (2009). Mantle shear-wave velocity structure beneath the Hawaiian hot spot. Science.

[56] Tarduno J., Bunge H.-P., Sleep N., Hansen U. (2009). The bent Hawaiian-emperor hotspot track: inheriting the mantle wind. Science.

[57] Tarduno J.A., Duncan R.A., Scholl D.W. (2003). The emperor seamounts: southward motion of the Hawaiian hotspot plume in earth's mantle. Science.

[58] Torsvik T.H., Doubrovine P.V., Steinberger B. (2017). Pacific plate motion change caused the Hawaiian-Emperor Bend. Nat. Commun..

[59] Bono R.K., Tarduno J.A., Bunge H.-P. (2019). Hotspot motion caused the Hawaiian-Emperor Bend and LLSVPs are not fixed. Nat. Commun..

[60] Sun W., Langmuir C.H., Ribe N.M. (2021). Plume-ridge interaction induced migration of the Hawaiian-Emperor seamounts. Sci. Bull..

[61] Burke K., Steinberger B., Torsvik T.H., Smethurst M.A. (2008). Plume generation zones at the margins of large low shear velocity provinces on the core-mantle boundary. Earth Planet Sci. Lett..

[62] Steinberger B., Torsvik T.H. (2012). A geodynamic model of plumes from the margins of large low shear velocity provinces. Geochem. Geophys. Geosyst..

[63] Hassan R., Müller R.D., Gurnis M. (2016). A rapid burst in hotspot motion through the interaction of tectonics and deep mantle flow. Nature.

[64] Courtillot V., Olson P. (2007). Mantle plumes link magnetic superchrons to phanerozoic mass depletion events. Earth Planet Sci. Lett..

[65] Sobolev S.V., Sobolev A.V., Kuzmin D.V. (2011). Linking mantle plumes, large igneous provinces and environmental catastrophes. Nature.

[66] Self S., Blake S., Sharma K. (2008). Sulfur and chlorine in late Cretaceous Deccan magmas and eruptive gas release. Science.

[67] Hofmann C., Courtillot V., Féraud G. (1997). Timing of the Ethiopian flood basalt event and implications for plume birth and global change. Nature.

[68] Sun W.-d., Hawkesworth C.J., Yao C. (2018). Carbonated mantle domains at the base of the Earth's transition zone. Chem. Geol..

