# Supplementary material for: An emerging plume head interacting with the Hawaiian plume tail
Source: Innovation (Camb). 2023 Feb 22;4(2):100404. doi: 10.1016/j.xinn.2023.100404 (PMC10005903; doi:10.1016/j.xinn.2023.100404)
Supplement: Document S1. Figures S1–S3, Tables S1 and S2, and supplemental information [file mmc1.pdf]

The Innovation, Volume *4*

Supplemental Information

An emerging plume head interacting with the Hawaiian plume tail

Lipeng Zhang, Zebin Cao, Robert E. Zartman, Congying Li, Saijun Sun, Lijun Liu, and Weidong Sun

1. Other double chains in the Pacific plate

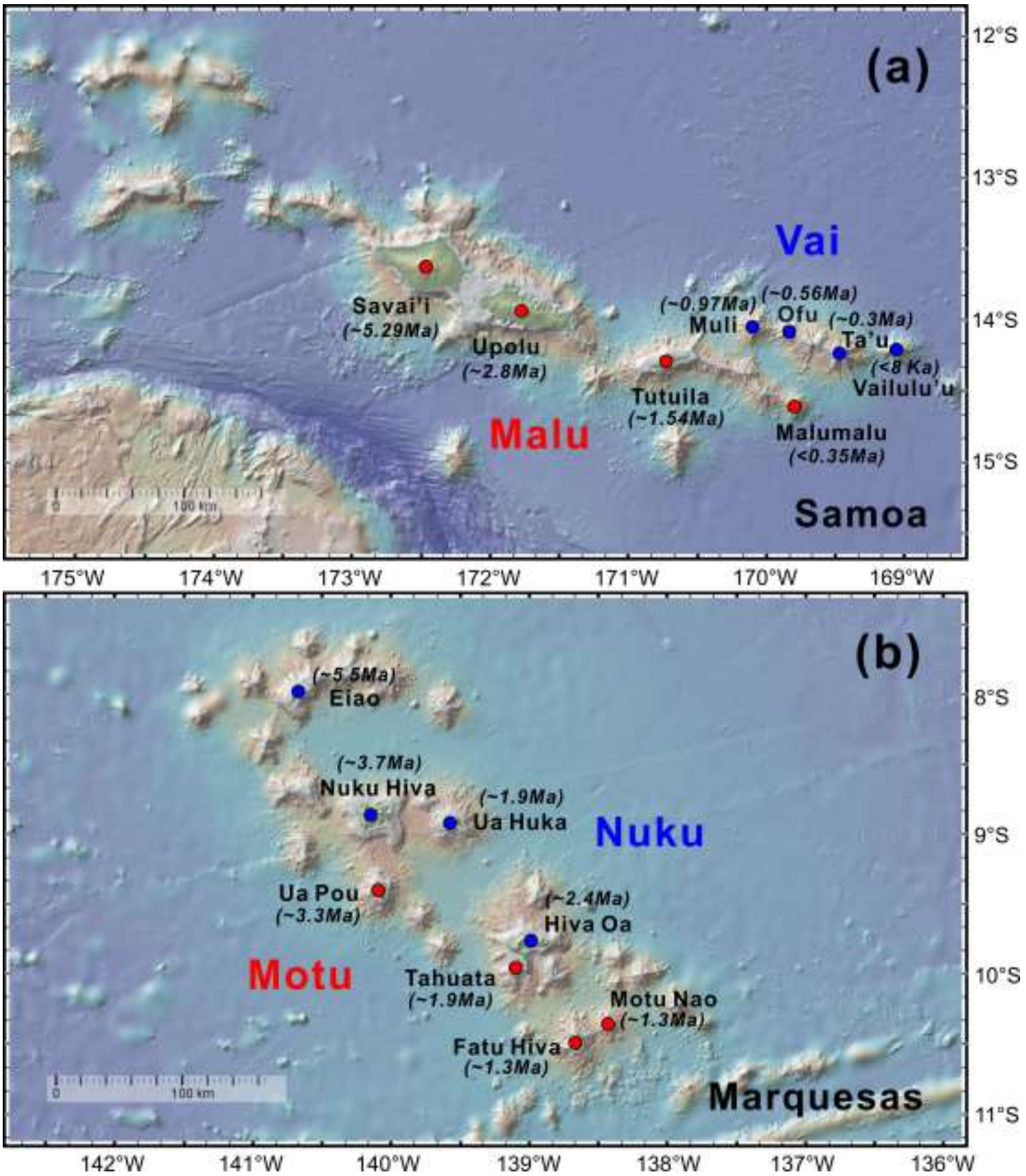

Figure S1 Map showing the other double volcano chains of **(a)** the Samoa and **(b)** the Marquesas plumes. In contrast to the Hawaiian plume, these two plumes are not bended nor increased in eruption. Also, these double chains appeared later than the Hawaiian double chain. The base maps are from the software of GeoMapApp. Age data are from literature as follow.

1. Jackson M, Hart S, Konter J, Kurz M, Blusztajn J, Farley K. Helium and lead isotopes reveal the geochemical geometry of the Samoan plume. *Nature* **514**, 355-

358 (2014).
2. Sims KW, et al. 238U-230Th-226Ra-210Pb-210Po, 232Th-228Ra, and 235U-231Pa constraints on the ages and petrogenesis of Vailulu'u and Malumalu Lavas, Samoa. *Geochemistry, Geophysics, Geosystems* **9**, (2008).
3. McDougall I. Age and evolution of the volcanoes of Tutuila, American Samoa. (1985).
4. Clouard V, Bonneville A. Ages of seamounts, islands, and plateaus on the Pacific plate. *Special Papers-Geological Society of America* **388**, 71 (2005).
5. Koppers AA, Russell JA, Jackson MG, Konter J, Staudigel H, Hart SR. Samoa reinstated as a primary hotspot trail. *Geology* **36**, 435-438 (2008).

2. Geochemical data for each island of the Hawaiian chain

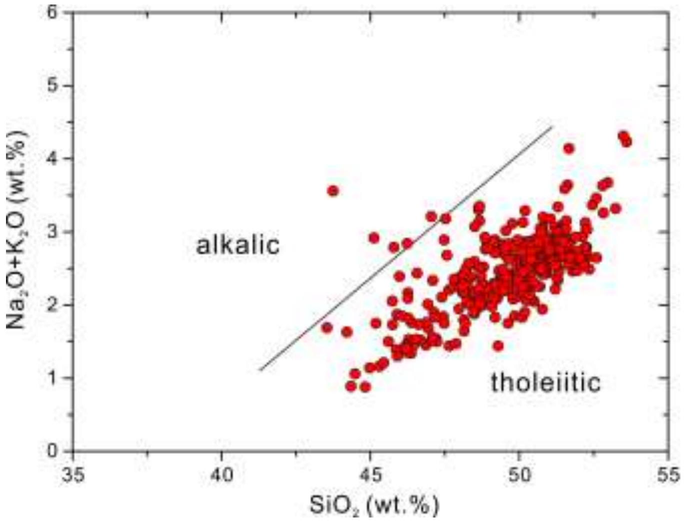

Figure S2 SiO_2 vs. total alkalis diagram. All the samples for which element concentrations are available were calculated as tholeiites. The tholeiitic/alkalic boundary is from Macdonald and Katsura (1964). The references used to determine the composition of each seamount are as follows.

Reference: Macdonald GA, Katsura T. Chemical Composition of Hawaiian Lavas1. *Journal of Petrology* **5**, (1964).

Data Sources for each island

Loihi

Abouchami, W., Hofmann, A.W., Galer, S.J., Frey, F.A., Eisele, J., Feigenson, M., 2005. Lead isotopes reveal bilateral asymmetry and vertical continuity in the Hawaiian mantle plume. Nature 434, 851.

Garcia, M.O., Foss, D.J.P., West, H.B., Mahoney, J.J., 1995. Geochemical and Isotopic Evolution of Loihi Volcano, Hawaii. Journal of Petrology 37, 1647-1674.

Garcia, M.O., Jorgenson, B.A., Mahoney, J.J., Ito, E., Irving, A.J., 1993. An evaluation of temporal geochemical evolution of Loihi Summit Lavas: Results from Alvin submersible dives. Journal of Geophysical Research Solid Earth 98, 537-550.

Mauna Loa

Abouchami, W., Galer, S.J.G., Hofmann, A.W., 2000. High precision lead isotope systematics of lavas from the Hawaiian Scientific Drilling Project. Chemical Geology 169, 187-209.

Blichert-Toft, J., Weis, D., Maerschalk, C., Agranier, A., Albarède, F., 2003. Hawaiian hot spot dynamics as inferred from the Hf and Pb isotope evolution of Mauna Kea volcano. Geochemistry Geophysics Geosystems 4.

Marske, J.P., Pietruszka, A.J., Weis, D., Garcia, M.O., Rhodes, J.M., 2007. Rapid passage of a small-scale mantle heterogeneity through the melting regions of Kilauea and Mauna Loa Volcanoes. Earth & Planetary Science Letters 259, 34-50.

Tanaka, R., Makishima, A., Nakamura, E., 2008. Hawaiian double volcanic chain triggered by an episodic involvement of recycled material: Constraints from temporal Sr–Nd–Hf–Pb isotopic trend of the Loa-type volcanoes. Earth & Planetary Science Letters 265, 450-465.

Tanaka, R., Nakamura, E., 2005. Boron isotopic constraints on the source of Hawaiian shield lavas. Geochimica et Cosmochimica Acta 69, 3385-3399.

Weis, D., Garcia, M.O., Rhodes, J.M., Jellinek, M., Scoates, J.S., 2011. Role of the deep mantle in generating the compositional asymmetry of the Hawaiian mantle plume. Nature Geoscience 4, 831-838.

Wanless, V.D., Garcia, M.O., Trusdell, F.A., Rhodes, J.M., Norman, M.D., Weis, D., Fornari, D.J., Kurz, M.D., Guillou, H., 2006. Submarine radial vents on Mauna Loa Volcano, Hawai'i. Geochemistry Geophysics Geosystems 7, 28.

Feigenson, M.D., Bolge, L.L., Carr, M.J., Herzberg, C.T., 2003. REE inverse modeling of HSDP2 basalts: Evidence for multiple sources in the Hawaiian plume. Geochemistry Geophysics Geosystems 4, 25.

Huailalai

Hanano, D., Weis, D., Scoates, J.S., Aciego, S., Depaolo, D.J., 2010. Horizontal and vertical zoning of heterogeneities in the Hawaiian mantle plume from the geochemistry of consecutive postshield volcano pairs: Kohala - Mahukona and Mauna Kea-Hualalai. Geochemistry Geophysics Geosystems 11.

Yamasaki, S., Kani, T., Hanan, B.B., Tagami, T., 2009. Isotopic geochemistry of Hualalai shield-stage tholeiitic basalts from submarine North Kona region, Hawaii. Journal of Volcanology & Geothermal Research 185, 223-230.

Kahoolawe

Abouchami, W., Hofmann, A.W., Galer, S.J., Frey, F.A., Eisele, J., Feigenson, M., 2005. Lead isotopes reveal bilateral asymmetry and vertical continuity in the Hawaiian mantle plume. Nature 434, 851.

Huang, S., Frey, F.A., Blichert-Toft, J., Fodor, R.V., Bauer, G.R., Xu, G., 2005. Enriched components in the Hawaiian plume: Evidence from Kahoolawe Volcano, Hawaii. Geochemistry Geophysics Geosystems 6.

Lanai

Abouchami, W., Hofmann, A.W., Galer, S.J., Frey, F.A., Eisele, J., Feigenson, M., 2005. Lead isotopes reveal bilateral asymmetry and vertical continuity in the Hawaiian mantle plume. Nature 434, 851.

Gaffney, A.M., Nelson, B.K., Blichert-Toft, J., 2005. Melting in the Hawaiian plume at 1-2 Ma as recorded at Maui Nui: The role of eclogite, peridotite, and source mixing. Geochemistry

Geophysics Geosystems 6.
Kilauea
Abouchami, W., Hofmann, A.W., Galer, S.J., Frey, F.A., Eisele, J., Feigenson, M., 2005. Lead isotopes reveal bilateral asymmetry and vertical continuity in the Hawaiian mantle plume. Nature 434, 851.
Marske, J.P., Pietruszka, A.J., Weis, D., Garcia, M.O., Rhodes, J.M., 2007. Rapid passage of a small-scale mantle heterogeneity through the melting regions of Kilauea and Mauna Loa Volcanoes. Earth & Planetary Science Letters 259, 34-50.
Tanaka, R., Makishima, A., Nakamura, E., 2008. Hawaiian double volcanic chain triggered by an episodic involvement of recycled material: Constraints from temporal Sr–Nd–Hf–Pb isotopic trend of the Loa-type volcanoes. Earth & Planetary Science Letters 265, 450-465.
Tanaka, R., Nakamura, E., 2005. Boron isotopic constraints on the source of Hawaiian shield lavas. Geochimica et Cosmochimica Acta 69, 3385-3399.
Hanyu T, Kimura J-I, Katakuse M, Calvert AT, Sisson TW, Naki Si., 2010. Source materials for inception stage Hawaiian magmas: Pb-He isotope variations for early Kilauea. Geochemistry Geophysics Geosystems 11.
Marske JP, Garcia MO, Pietruszka AJ, Rhodes JM, Norman MD., 2008. Geochemical variations during Kilaueas Pu'u eruption reveal a fine-scale mixture of mantle heterogeneities within the Hawaiian plume. Journal of Petrology 49, 1297-1318.
Pietruszka AJ, Norman MD, Garcia MO, Marske JP, Burns DH., 2013. Chemical heterogeneity in the Hawaiian mantle plume from the alteration and dehydration of recycled oceanic crust. Earth and Planetary Science Letters 361, 298-309.
Mauna Kea
Abouchami, W., Galer, S.J.G., Hofmann, A.W., 2000. High precision lead isotope systematics of lavas from the Hawaiian Scientific Drilling Project. Chemical Geology 169, 187-209.
Abouchami, W., Hofmann, A.W., Galer, S.J., Frey, F.A., Eisele, J., Feigenson, M., 2005. Lead isotopes reveal bilateral asymmetry and vertical continuity in the Hawaiian mantle plume. Nature 434, 851.
Blichert-Toft, J., Weis, D., Maerschalk, C., Agranier, A., Albarède, F., 2003. Hawaiian hot spot dynamics as inferred from the Hf and Pb isotope evolution of Mauna Kea volcano. Geochemistry Geophysics Geosystems 4.
Eisele, J., Abouchami, W., Galer, S.J.G., Hofmann, A.W., 2003. The 320 kyr Pb isotope evolution of Mauna Kea lavas recorded in the HSDP‐2 drill core. Geochemistry Geophysics Geosystems 4.
Hanano, D., Weis, D., Scoates, J.S., Aciego, S., Depaolo, D.J., 2010. Horizontal and vertical zoning of heterogeneities in the Hawaiian mantle plume from the geochemistry of consecutive postshield volcano pairs: Kohala‐Mahukona and Mauna Kea-Hualalai. Geochemistry Geophysics Geosystems 11.
Rhodes, J.M., Vollinger, M.J., 2004. Composition of basaltic lavas sampled by phase‐2 of the Hawaii Scientific Drilling Project: Geochemical stratigraphy and magma types. Geochemistry Geophysics Geosystems 5, -.
Silva, I.G.N., Weis, D., Scoates, J.S., 2013. Isotopic systematics of the early Mauna Kea shield phase and insight into the deep mantle beneath the Pacific Ocean. Geochemistry Geophysics Geosystems 14, 659-676.

Huang, S., Frey, F.A., 2003. Trace element abundances of Mauna Kea basalt from phase 2 of the Hawaii Scientific Drilling Project: Petrogenetic implications of correlations with major element content and isotopic ratios. Geochemistry Geophysics Geosystems 4, 43.

Kohala

Abouchami, W., Hofmann, A.W., Galer, S.J., Frey, F.A., Eisele, J., Feigenson, M., 2005. Lead isotopes reveal bilateral asymmetry and vertical continuity in the Hawaiian mantle plume. Nature 434, 851.

Hanano, D., Weis, D., Scoates, J.S., Aciego, S., Depaolo, D.J., 2010. Horizontal and vertical zoning of heterogeneities in the Hawaiian mantle plume from the geochemistry of consecutive postshield volcano pairs: Kohala - Mahukona and Mauna Kea-Hualalai. Geochemistry Geophysics Geosystems 11,

Haleakala

Phillips, E.H., Sims, K.W.W., Sherrod, D.R., Salters, V.J.M., Blusztajn, J., Dulai, H., 2016. Isotopic constraints on the genesis and evolution of basanitic lavas at Haleakala, Island of Maui, Hawaii. Geochimica et Cosmochimica Acta 195, 201-225.

West Maui

Gaffney, A.M., Nelson, B.K., Blicherttoft, J., 2004. Geochemical Constraints on the Role of Oceanic Lithosphere in Intra-Volcano Heterogeneity at West Maui, Hawaii. Journal of Petrology 45, 1663-1687(1625).

East Molokai

Xu, G., Frey, F.A., Clague, D.A., Weis, D., Beeson, M.H., 2005. East Molokai and other Kea - trend volcanoes: Magmatic processes and sources as they migrate away from the Hawaiian hot spot. Geochemistry Geophysics Geosystems 6.

West Molokai

Xu, G., Frey, F.A., Clague, D.A., Abouchami, W., Blichert-Toft, J., Cousens, B., Weisler, M., 2007. Geochemical characteristics of West Molokai shield- and postshield-stage lavas: Constraints on Hawaiian plume models. Geochemistry Geophysics Geosystems 8.

Koolau

Abouchami, W., Hofmann, A.W., Galer, S.J., Frey, F.A., Eisele, J., Feigenson, M., 2005. Lead isotopes reveal bilateral asymmetry and vertical continuity in the Hawaiian mantle plume. Nature 434, 851.

Fekiacova, Z., Abouchami, W., Galer, S.J.G., Garcia, M.O., Hofmann, A.W., 2007. Origin and temporal evolution of Ko‘olau Volcano, Hawai‘i: Inferences from isotope data on the Ko‘olau Scientific Drilling Project (KSDP), the Honolulu Volcanics and ODP Site 843. Earth & Planetary Science Letters 261, 65-83.

Roden, M.F., Trull, T., Hart, S.R., Frey, F.A., 1994. New He, Nd, Pb, and Sr isotopic constraints on the constitution of the Hawaiian plume: Results from Koolau Volcano, Oahu, Hawaii, USA. Geochimica et Cosmochimica Acta 58, 1431-1440.

Salters, V.J.M., Blichert-Toft, J., Fekiacova, Z., Sachi-Kocher, A., Bizimis, M., 2006. Isotope and trace element evidence for depleted lithosphere in the source of enriched Ko’olau basalts. Contributions to Mineralogy & Petrology 151, 297-312.

Tanaka, R., Makishima, A., Nakamura, E., 2008. Hawaiian double volcanic chain triggered by an episodic involvement of recycled material: Constraints from temporal Sr–Nd–Hf–Pb isotopic trend of the Loa-type volcanoes. Earth & Planetary Science Letters 265, 450-465.

Tanaka, R., Nakamura, E., Takahashi, E., 2002. Geochemical evolution of Koolau Volcano, Hawaii. Hawaiian Volcanoes: Deep Underwater Perspectives 128, 311-332.

Haskins EH, Garcia MO., 2004. Scientific drilling reveals geochemical heterogeneity within the Ko'olau shield, Hawai'i. Contributions to Mineralogy and Petrology 147, 162-188.

Huang S, Frey FA., 2005. Recycled oceanic crust in the Hawaiian Plume: evidence from temporal geochemical variations within the Koolau Shield. Contributions to Mineralogy & Petrology 149, 556-575.

Waianae

Coombs, M., Clague, D.A., Moore, J.G., Cousens, B.L., 2004. Growth and collapse of Waianae Volcano, Hawaii, as revealed by exploration of its submarine flanks. Geochem. Geophys. Geosyst. 5, Q08006.

Van der Zander, I., Sinton, J.M., Mahoney, J.J., 2010. Late shield-stage silicic magmatism at Wai‘anae volcano: Evidence for hydrous crustal melting in Hawaiian volcanoes. Journal of Petrology 51, 671-701.

Williamson, N.M.B., Weis, D., Scoates, J.S., Pelletier, H., Garcia, M.O., 2019. Tracking the Geochemical Transition Between the Kea-Dominated Northwest Hawaiian Ridge and the Bilateral Loa-Kea Trends of the Hawaiian Islands. Geochemistry Geophysics Geosystems 20, 4354-4369.

Kauai

Garcia, M.O., Swinnard, L., Weis, D., Greene, A.R., Tagami, T., Sano, H., Gandy, C.E., 2010. Petrology, Geochemistry and Geochronology of Kaua‘i Lavas over 4.5 Myr: Implications for the Origin of Rejuvenated Volcanism and the Evolution of the Hawaiian Plume. Journal of Petrology 51, 1507-1540.

Mukhopadhyay, S., Lassiter, J.C., Farley, K.A., Bogue, S.W., 2003. Geochemistry of Kauai shield-stage lavas: Implications for the chemical evolution of the Hawaiian plume. Geochemistry Geophysics Geosystems 4, 32.

Williamson, N.M.B., Weis, D., Scoates, J.S., Pelletier, H., Garcia, M.O., 2019. Tracking the Geochemical Transition Between the Kea-Dominated Northwest Hawaiian Ridge and the Bilateral Loa-Kea Trends of the Hawaiian Islands. Geochemistry Geophysics Geosystems 20, 4354-4369.

Nihoa

Harrison, L.N., Weis, D., Garcia, M.O., 2017. The link between Hawaiian mantle plume composition, magmatic flux, and deep mantle geodynamics. Earth and Planetary Science Letters 463, 298-309.

Harrison, L.N., Weis, D., 2018. The Size and Emergence of Geochemical Heterogeneities in the Hawaiian Mantle Plume Constrained by Sr-Nd-Hf Isotopic Variation Over similar to 47 Million Years. Geochemistry Geophysics Geosystems 19, 2823-2842.

Northwest Hawaiian Rigde

Harrison, L.N., Weis, D., 2018. The Size and Emergence of Geochemical Heterogeneities in the Hawaiian Mantle Plume Constrained by Sr-Nd-Hf Isotopic Variation Over similar to 47 Million Years. Geochemistry Geophysics Geosystems 19, 2823-2842.

Harrison, L.N., Weis, D., Garcia, M.O., 2017. The link between Hawaiian mantle plume composition, magmatic flux, and deep mantle geodynamics. Earth and Planetary Science Letters 463, 298-309.

Emperor Chain

Regelous, M., Hofmann, A.W., Abouchami, W., Galer, S.J.G., 2003. Geochemistry of lavas from the Emperor Seamounts, and the geochemical evolution of Hawaiian magmatism from 85 to 42 Ma. Journal of Petrology 44, 113-140(128).

Keller, R.A., Fisk, M.R., White, W.M., 2000. Isotopic evidence for Late Cretaceous plume-ridge interaction at the Hawaiian hotspot. Nature 405, 673-676.

Garcia, M.O., Smith, J.R., Tree, J.P., Weis, D., Harrison, L., Jicha, B.R., 2015. "Petrology, geochemistry, and ages of lavas from Northwest Hawaiian Ridge volcanoes", The Origin, Evolution, and Environmental Impact of Oceanic Large Igneous Provinces, Clive R. Neal, William W. Sager, Takashi Sano, Elisabetta Erba

Tree, J., 2016. Mantle Potential Temperatures of 4.5 to 47 Ma Hawaiian Volcanoes Using Olivine Thermometry: Implications for Melt Flux Variations. A master's thesis of the University of Hawaiʻi at Mānoa

Ontong Java

Tejada, M.L.G., Mahoney, J.J., Castillo, P.R., Ingle, S.P., Sheth, H.C., Weis, D., 2004. Pin-pricking the elephant: evidence on the origin of the Ontong Java Plateau from Pb-Sr-Hf-Nd isotopic characteristics of ODP leg 192 basalts. Origin and Evolution of the Ontong Java Plateau 229, 133-150.

Tejada, M.L.G., Mahoney, J.J., Neal, C.R., Duncan, R.A., Petterson, M.G., 2002. Basement geochemistry and geochronology of Central Malaita, Solomon Islands, with implications for the origin and evolution of the Ontong Java Plateau. Journal of Petrology 43, 449-484.

Tejada, M.L.G., Suzuki, K., Hanyu, T., Mahoney, J.J., Ishikawa, A., Tatsumi, Y., Chang, Q., Nakai, S., 2013. Cryptic lower crustal signature in the source of the Ontong Java Plateau revealed by Os and Hf isotopes. Earth and Planetary Science Letters 377-378, 84-96.

Manihiki Plateau

Hoernle, K., Hauff, F., van den Bogaard, P., Werner, R., Mortimer, N., Geldmacher, J., Garbe-Schönberg, D., Davy, B., 2010. Age and geochemistry of volcanic rocks from the Hikurangi and Manihiki oceanic Plateaus. Geochimica Et Cosmochimica Acta 74, 7196-7219.

Ingle, S., Mahoney, J.J., Sato, H., Coffin, M., Kimura, J.I., Hirano, N., Nakanishi, M., 2007. Depleted mantle wedge and sediment fingerprint in unusual basalts from the Manihiki Plateau, central Pacific Ocean. Geology 35, 595-598.

Mahoney, J.J., Spencer, K.J., 1991. Isotopic evidence for the origin of the Manihiki and Ontong Java oceanic plateaus. Earth and Planetary Science Letters 104, 196-210.

Timm, C., Hoernle, K., Werner, R., Hauff, F., den Bogaard, P.v., Michael, P., Coffin, M.F., Koppers, A., 2011. Age and geochemistry of the oceanic Manihiki Plateau, SW Pacific: New evidence for a plume origin. Earth and Planetary Science Letters 304, 135-146.

Hikurangi Plateau

Hoernle, K., Hauff, F., van den Bogaard, P., Werner, R., Mortimer, N., Geldmacher, J., Garbe-Schönberg, D., Davy, B., 2010. Age and geochemistry of volcanic rocks from the Hikurangi and Manihiki oceanic Plateaus. Geochimica Et Cosmochimica Acta 74, 7196-7219.

3. Geodynamic model setup

To study plume-plume interaction, we used a user-updated version of 3D spherical finite element code CitcomS. On finite-element mesh, we solved the conservation equations of mass, momentum, and energy, under the Boussinesq approximation:

$$\nabla \cdot \vec{u} = 0$$

$$-\nabla P + \nabla \cdot [\eta(\nabla \vec{u} + \nabla^T \vec{u})] + \rho_m \alpha \Delta T \vec{g} = 0$$

$$\frac{\partial T}{\partial t} + \vec{u} \cdot \nabla T = \kappa \nabla^2 T$$

where $\vec{u}$ is velocity, P is dynamic pressure, η is effective viscosity, ρ_m is reference mantle density, α is thermal expansion coefficient, ΔT is thermal anomaly, $\vec{g}$ is gravitational acceleration, T is temperature, and κ is thermal diffusivity, respectively.

The model spans 30° × 30° × 2000 km in longitude × latitude × radius, a region much larger than the Hawaiian volcanic chain. The vertical and horizontal resolution are 15 km and 6 km, respectively. Passive tracers are used to track the evolution of two plumes, assuming no extra chemical buoyancy of plume material.

Boundary conditions involve temperature and velocity boundary conditions at surface. The surface potential temperature is set to be 0 ˚C, beneath the surface we set up a thermal oceanic lithosphere based on half-space cooling model, assuming 80 Myr old seafloor. The surface has been prescribed a constant westward plate motion at 10 cm/yr through the whole simulation.

To model realistic plume structure above 1000 km depth, we fixed heat sources as cones at depth from 1400 km to 1500 km. The resultant plume conduits have spatial averaged excess temperatures varying from 100 ˚C to 300 ˚C at LAB depth (~100 km). The present plume conduit under Hawaii has a spatial averaged excess temperature around 300 ˚C, which is consistent with literature estimates[5,6]. The heat source for Loa trend is located 220 km south to the one for Kea trend, and its base radius is twice as large as that for Kea trend. To simulate the evolution of Hawaiian volcanic chain, we started with the heat source for Kea trend only. After 40 Myr, the Kea trend is formed and became steady. At this time, the heat source for Loa trend was added (Figure S3).

Temperature- and depth-dependent Newtonian rheology is applied for the whole model domain. The background mantle viscosity at the ambient mantle temperature has a 3 layer-profile: lithosphere (0-100km), asthenosphere (100-410 km), and below (410-2000 km). Their respective

viscosity values are 0.1*η_0 (10^{20} Pa s), 0.1*η_0 (10^{20} Pa s), η_0 (10^{21} Pa s), where η_0 is the reference viscosity (10^{21} Pa s). The temperature-dependent Newtonian rheology follows:

$$\eta = \eta_{b.g.}(\mathrm{r})\exp(\frac{E(r)}{T+T_0} - \frac{E(r)}{T_m+T_0})$$

where η is effective viscosity, $\eta_{b.g.}$ is background mantle viscosity, E is activation energy, T is temperature, T_0 is temperature offset, and T_m is ambient mantle temperature. The activation energy used in this study is the same as in Hu et al. (2018). With strong temperature-dependent Newtonian rheology, our strong lithosphere reaches 10^{23} Pa s in the upper part and smoothly transient to weak asthenosphere. Other physical parameters used are shown in table S2.

References:

1. Li, X., Kind, R., Priestley, K., Sobolev, S. V., Tilmann, F., Yuan, X., & Weber, M. (2000). Mapping the Hawaiian plume conduit with converted seismic waves. Nature, 405(6789), 938-941.
2. Putirka, K. (2008). Excess temperatures at ocean islands: Implications for mantle layering and convection. Geology, 36(4), 283-286.
3. Hu, J., Liu, L., & Zhou, Q. (2018). Reproducing past subduction and mantle flow using high‐resolution global convection models. Earth and Planetary Physics, 2(3), 189-207.

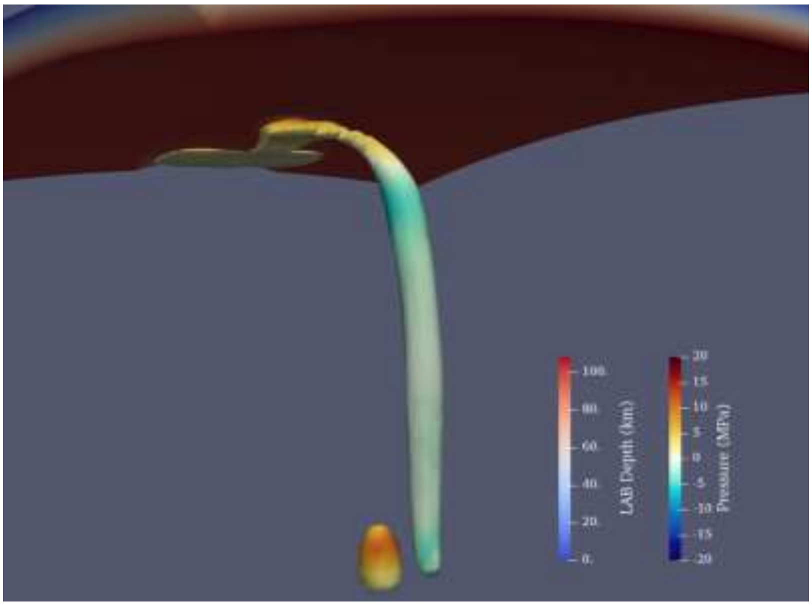

Figure S3 3D view of the Hawaiian plume system at 40 Myr from starting time, when the Loa plume emerged from the lower mantle. View towards northwest direction. The right plume is established Kea plume, the left one is newly formed Loa plume which has a larger size than the Kea plume. Plumes are presented by isothermal surface with 50 ˚C excess temperature.

Table S2 Physical parameters used in geodynamic model

Parameter	Symbol	Value	Unit
Earth radius	R	6371.0	km
Gravitational acceleration	g	9.81	m/s^2
Reference mantle density	ρ	3340	kg/m^3
Reference viscosity	η_0	10^{21}	Pa s
Background viscosity	$\eta_{b.k.}$	10^{20}, 10^{20}, 10^{21}	Pa s
Thermal diffusivity	κ	10^{-6}	m^2/s
Thermal expansivity	α	3.0×10^{-5}	1/°C
Rayleigh number	Ra	5.0e8	-
Activation energy	E	100, 166, 100	kJ/mol
Minimum viscosity	η_{min}	1.0e19	Pa s
Maximum viscosity	η_{max}	1.0e23	Pa s

4. Migration history of plume system center

We identified the centers of Hawaiian plume system in our geodynamic model at 200 km depth which can be used to show the migration of surface volcanic activities. The hottest part of the Hawaiian plume system is defined as the center of the plume system at each output timestep. Since melting processes highly depend on the P-T condition, the hottest part may well represent the center of the plume system with the highest melt production rate at this depth. After identification, the centers were assumed to move with the oceanic lithosphere at the same speed. The temporal and spatial evolution of plume system center is shown in Figure 5 in the main text.
