# Supplementary material for: An emerging plume head interacting with the Hawaiian plume tail
Source: Innovation (Camb). 2023 Feb 22;4(2):100404. doi: 10.1016/j.xinn.2023.100404 (PMC10005903; doi:10.1016/j.xinn.2023.100404)
Supplement: Document S2. Article plus supplemental information [file mmc2.pdf]

www.the-innovation.org

ARTICLE

An emerging plume head interacting with the Hawaiian plume tail

Lipeng Zhang,[1,4] Zebin Cao,[3] Robert E. Zartman,[1] Congying Li,[1,2] Saijun Sun,[1,4] Lijun Liu,[3,*] and Weidong Sun[1,2,5,*]

*Correspondence: ljliu@illinois.edu (L.L.); weidongsun@qdio.ac.cn (W.S.)

Received: November 11, 2022; Accepted: February 16, 2023; Published Online: February 22, 2023; https://doi.org/10.1016/j.xinn.2023.100404

© 2023 The Author(s). This is an open access article under the CC BY license (http://creativecommons.org/licenses/by/4.0/).

GRAPHICAL ABSTRACT

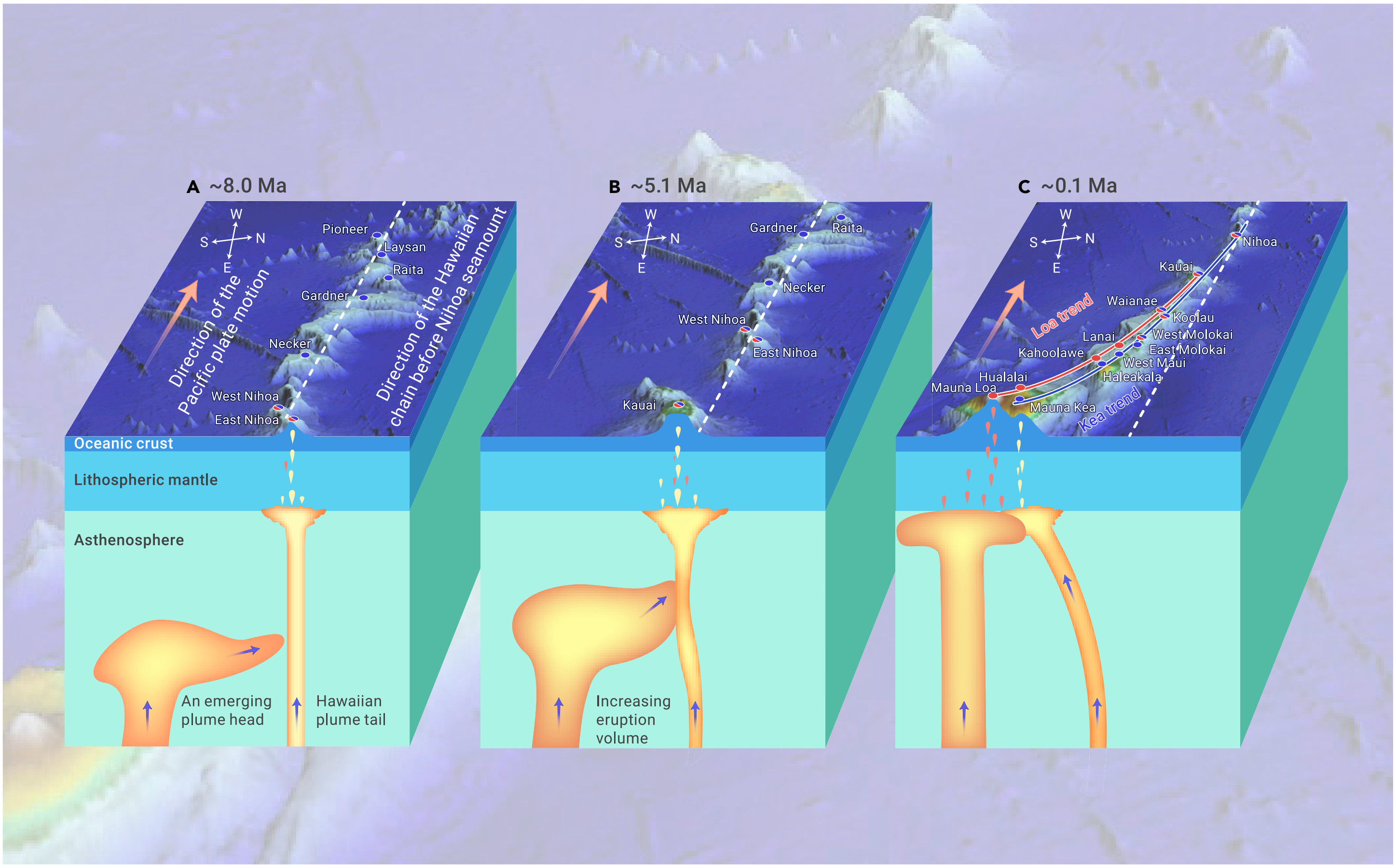

PUBLIC SUMMARY

- Hawaiian hotspot track displays two subparallel isotopic trends since 5 Ma.
- This is coeval with increased eruption rate and southward bending of the track.
- These data support a new plume head emerging next to the old Hawaiian plume.
- Our double-plume model may represent a new mechanism for hotspot evolution.

The Innovation

An emerging plume head interacting with the Hawaiian plume tail

Lipeng Zhang,[1,4] Zebin Cao,[3] Robert E. Zartman,[1] Congying Li,[1,2] Saijun Sun,[1,4] Lijun Liu,[3,*] and Weidong Sun[1,2,5,*]

[1]Center of Deep Sea Research, Institute of Oceanology, Chinese Academy of Sciences, Qingdao 266071, China

[2]Deep-Sea Multidisciplinary Research Center, Laoshan Laboratory, Qingdao 266237, China

[3]Department of Geology, University of Illinois at Urbana-Champaign, Urbana, IL 61820, USA

[4]Laboratory for Marine Geology, Laoshan Laboratory, Qingdao 266237, China

[5]University of the Chinese Academy of Sciences, Beijing 100049, China

*Correspondence: ljliu@illinois.edu (L.L.); weidongsun@qdio.ac.cn (W.S.)

Received: November 11, 2022; Accepted: February 16, 2023; Published Online: February 22, 2023; https://doi.org/10.1016/j.xinn.2023.100404

© 2023 The Author(s). This is an open access article under the CC BY license (http://creativecommons.org/licenses/by/4.0/).

Citation: Zhang L., Cao Z., Zartman R.E., et al., (2023). An emerging plume head interacting with the Hawaiian plume tail. The Innovation **4(2)**, 100404.

The Hawaiian-Emperor seamount chain has shown two subparallel geographical and geochemical volcanic trends, Loa and Kea, since ∼5 Ma, for which numerous models have been proposed that usually involve a single mantle plume sampling different compositional sources of the deep or shallow mantle. However, both the dramatically increased eruption rate of the Hawaiian hotspot since ∼5 Ma and the nearly simultaneous southward bending of the Hawaiian chain remain unexplained. Here, we propose a plume-plume interaction model where the compositionally depleted Kea trend represents the original Hawaiian plume tail and the relatively enriched Loa trend represents an emerging plume head southeast of the Hawaiian plume tail. Geodynamic modeling further suggests that the interaction between the existing Hawaiian plume tail and the emerging Loa plume head is responsible for the southward bending of the Hawaiian chain. We show that the arrival of the new plume head also dramatically increases the eruption rate along the hotspot track. We suggest that this double-plume scenario may also represent an important mechanism for the formation of other hotspot tracks in the Pacific plate, likely reflecting a dynamic reorganization of the lowermost mantle.

INTRODUCTION

The conventional plume hypothesis predicts that a single chain of volcanos with age progression should form as a plate passes over a relatively stationary mantle plume.[1,2] The Hawaiian-Emperor chain, however, does not strictly follow this prediction. The volcanos of the Hawaiian chain progressively bifurcated into two subparallel geographical and geochemical trends since ∼5 Ma: the Loa and the Kea trends (Figure 1). These two trends are of significant compositional differences in major and trace elements and are best illustrated by the contrasting Pb and Nd isotopes. Overall, the Loa trend has lower ε_{Nd} and higher $^{208}Pb^*/^{206}Pb^*$ compared with the Kea trend and with mid-ocean ridge basalts (Figures 1 and 2).[3–16] The two isotopically distinct trends of the Hawaiian hotspot were interpreted previously using a variety of models.[5–8,13,16–22] For example, it has been suggested that the Hawaiian plume is concentrically zoned in chemical and isotopic compositions, with the Loa trend sampling the center and the Kea trend sampling the margins.[17,23,24] Based on high-precision Pb isotopes, it is proposed that the two trends have very little compositional overlap such that they originated from bilateral, non-concentric plume zones.[5,15,25] The geochemical differences between the Kea and Loa trends were also explained by two distinct sources at the core-mantle boundary.[6,8,11,13,14,16] Another study proposed that the chemical variation of the Hawaiian mantle plume was controlled by its thermal structure.[7] A recent study attributed the double trends to a recent azimuthal change in the motion of the Pacific plate such that the Loa trend sampled the shallow portions of the plume dominated by a pyroxenite melt zone, while the Kea trend sampled the deep peridotite melt zone at the center of the Hawaii plume.[18] However, the drift direction of the Pacific plate has changed multiple times in the geological history, but these changes do not always correlate with subparallel hotspot chains showing distinct geochemical characteristics within the Pacific plate. In brief, none of the published models could explain the fact that the bifurcated geographical and geochemical trends did not appear until ∼5 Ma, a phenomenon not only observed in Hawaii (Figures 1 and 2) but also in other hotspot tracks (Figure S1).

RESULTS AND DISCUSSION

The synchronous occurrence of geochemical bifurcation, bending of the seamount chain, and rapidly increasing eruption rates

According to geochemical data, the Loa trend began to appear in the Nihoa seamount (∼8 Ma)[16] and became clearly discernable in the Kauai seamount (∼5 Ma).[5,6,8,13,14,16,29] To the west of the Nihoa seamount, the Hawaiian chain, including the entire Emperor chain, is classified as belonging to the Kea trend (Figures 1 and 2). Therefore, the Kea trend represents the original Hawaiian plume tail (Figure 1) that has lasted for more than 80 Myr, whereas the Loa trend should be a new one (<8 Myr)[8,13,30] with enriched isotope compositions, which are typical for plume heads in the Pacific plate, such as Ontong Java, Manihiki, and Hikurangi (Figure 2).[31–37] Some researchers found that some samples from the Daikakuji seamount have enriched geochemical signatures.[13,14,38,39] This is at least partially due to alterations.[39] Furthermore, their major and trace element and isotope compositions show obvious differences from those of the Loa trend.[38,39] Especially for the most representative Pb isotope of the Loa trend, the Daikakuji samples do not lie exactly on the main field of the Loa trend (Figure 2). It erupted near the Hess Rise and thus may have been influenced by underplated enriched magmas from the southern Hess Rise, which has high radiogenic Pb isotopes and low Nd isotopes.[31] Previous studies have also attributed these isolated isotope characteristics of Daikakuji to the presence of a small mantle heterogeneity,[13,14] which is essentially different from the seamounts (<8 Ma) of the Loa trend.

Remarkably, the occurrence of bifurcated geochemical characteristics in the Hawaiian magmas in <8 Ma is synchronous with the abrupt bending of the hotspot chain (Figure 1). Starting roughly at ∼8–5 Ma, the Hawaiian chain bends to the south by ∼45° from its original trajectory (defined by the Northwest Hawaiian Ridge), with a total offset of ∼280 km by now (Figure 1). This was attributed to the recent azimuthal change in Pacific plate motion.[18] However, no such bent has been observed in other seamount chains on the Pacific plate during the same period (Figure S1), which precludes the changing plate motion as the cause.

According to the classical plume hypothesis, a mantle plume is characterized by a large head that usually forms a large igneous province at the surface, followed by a long, thin tail that corresponds to a volcano chain.[1,2,40–46] The Hawaiian-Emperor volcano chain is generally attributed to the tail of the Hawaiian plume. However, the eruption rate of the Hawaiian plume has increased dramatically in the last few million years (Figure 1).[27,47,48] The main island of Hawaii, with an estimated volume of ∼0.21 million cubic kilometers,[48] is much larger than volcanos within most other hotspot chains. The average eruption rate of the Hawaiian plume was mostly ∼0.017 km^3/year between 47.5 and 5 Ma. This suddenly increased to ∼0.05 km^3/year at ∼5 Ma at Kauai,[48] contemporaneous with the commencement of the southward bending (Figure 1). The total eruption rate of the Hawaiian plume sharply increased again to ∼0.66 km^3/year (i.e., ∼21 m^3/s) at the latest stage,[26,27] ∼40 times higher than the average eruption rate between 47.5 and 5 Ma and even comparable to that of a plume head. For example, the Deccan Trap,[49] a well-studied large igneous province erupted from an underlying plume head, has a total volume of ∼0.7 million cubic kilometers,[1] erupted within one million years, which corresponds to an average eruption rate of ∼0.7 km^3/year. It is followed by a thin island chain to the south, formed by the eruption of the Reunion mantle plume.[50,51] The distinctive eruption behavior of the Hawaiian plume seems to contradict the mantle plume hypothesis, which

www.the-innovation.org

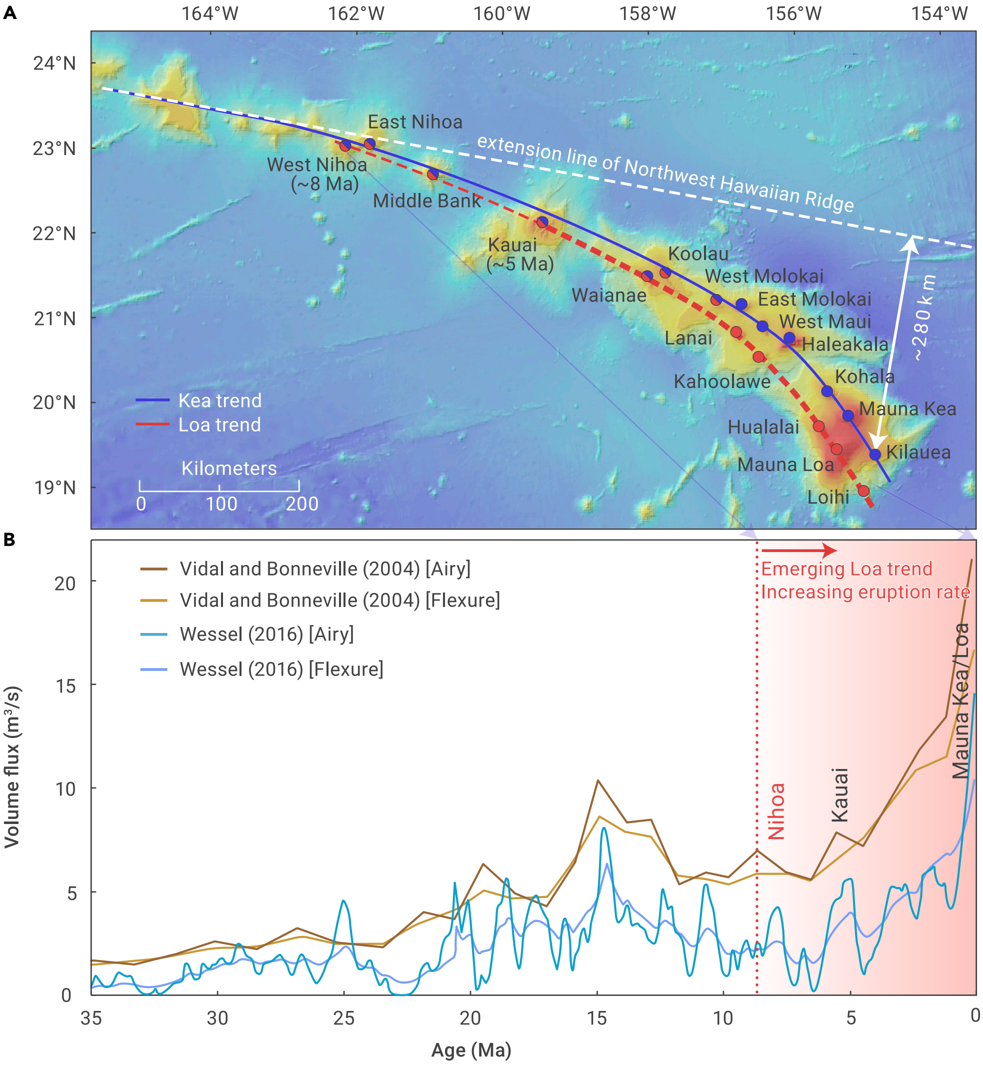

Figure 1. Map showing double chains of the Hawaiian hotspot The bending of the Hawaiian chains started at ~8 Ma. The Kea trend (north) is the original Hawaiian chain, whereas the Loa trend (south) is newly emerged as indicated by isotopic characteristics (see Figure 2). The Nihoa seamount (~8 Ma) is the first volcano that has samples with geochemical signatures of the Loa trend, and it became clearly discernable at Kauai (~5 Ma). Correspondingly, the volume and magma flux of the Hawaiian chain has increased dramatically since ~5 Ma (Figure 1B). Volume flux is estimated based on the airy and flexural compensation models.[26,27] The latest eruption rate is ~21 m^3/s, i.e., 0.66 $km^3/year$. Interestingly, double chains are also reported for the Samoa and the Marquesas volcano chains (Figure S1). Geochronology data from previous literature of the seamounts are compiled in the supplemental information.

predicts that the eruption rate of the plume tail should be significantly lower than that of the plume head.[1,13,52] It is important to note that the rapidly increasing eruption rate of the Hawaiian plume in the last few million years is due to the growth of the Loa trend, which became clearly discernable at ~5 Ma (Figures 1 and 2).

An emerging plume head interacting with the original Hawaiian plume

All these observations can be better explained by the interaction of an emerging plume head with the waning Hawaiian plume tail. We name this new plume the Loa plume. The root of the Loa plume was located to the southeast of that of the Hawaiian plume, and the subsequent southward migration of the hotspot chain, the increasing magma eruption rates, and the progressive separation of the two geochemical trends reflect the dynamic interaction and competition of the two plumes as they strive to dominate the source of surface volcanism. Nihoa, which erupted ~8 Ma, provides the first compositional signal for a different mantle source (Figures 1 and 2), which we interpret as representing the arrival of the Loa plume head to the mantle melt zone.

To better understand this plume-plume interaction, we designed a high-resolution 3D geodynamic model using the code CitcomS.[53] The model starts with an established Hawaiian plume conduit that has a diameter of 100 km and a growing Loa plume head whose conduit is twice as wide and 250 km further south, both rooted at 1,500 km depth. More model details can be found in the supplemental information. We track the movement of the Loa plume head as it approaches the surface and interacts with the Hawaiian plume.

As a plume ascends, the upper half of the plume head and the region above experience positive dynamic pressure due to upward compression. The lower plume head and the region further below experience negative dynamic pressure due to extension. Because the nearby Hawaiian plume produces a conduit of negative dynamic pressure throughout the mantle (Figure 3A), the rising Loa plume head is initially attracted by the Hawaiian plume conduit following the lateral pressure gradient (Figures 3B and 3C). Prior to the arrival of the Loa plume, the Hawaiian conduit is entrained northwestward following the Pacific plate motion (Figures 3A and S3). Due to the larger buoyancy than that of the Hawaiian tail, the Loa plume head rises faster and more vertically. In addition, the temporally reduced mantle viscosity due to increased temperature helps alleviate plate entrainment so that the location of the Loa plume head around the lithosphere-asthenosphere boundary (LAB) jumped eastward by ~200 km, leading to a clear gap of plume flux along the direction of plate motion. Consequently, this creates a magmatic hiatus along the Hawaiian conduit. In observation, there was a ~200 km wide gap in the Hawaiian chain without seamounts to the west of Kauai when the bending started (Figure 1). This is consistent with the modeled plume-plume interaction (Figure 4).

Because the strong lateral pressure gradient diverts the Loa plume head toward the Hawaiian conduit, the Loa plume initially reaches the LAB at a similar location as the original Hawaiian conduit, which is aligned with the earlier Hawaiian chain downstream of the Pacific motion direction (Figure 3). Initial mixing of the two plumes implies that magmas with Loa characteristics should start to appear along the original Hawaiian chain, as is observed starting in Nihoa, and the islands/seamounts from Nihoa to West Molokai display both Loa and Kea geochemical signatures before the distinct Loa trend has fully emerged (Figure 1). Their geochemical compositions vary dramatically, plotted on both the Loa and Kea trends (Figure 2), which clearly indicate "mixing" of the Kea and Loa magmas, and also with the depleted mantle.[8,17] The highest $^{208}Pb^{*}/^{206}Pb^{*}$ and lowest ε_{Nd} values among all Hawaiian basalts are seen in Koolau, implying that these Koolau samples are close to the Loa end member (Figure 2). However, the trace elements in inclusions of Koolau basalts are highly varied and show geochemical characteristics of both Kea and Loa.[7]

After the plume head settles down in the asthenosphere, its upward motion slows down. Accordingly, the positive dynamic pressure within the plume head decreases rapidly (Figures 3D and 3E). Consequently, the reduced lateral pressure difference from that of the Hawaiian conduit (the Kea trend) means that the Loa plume is driven mostly by its own buoyancy and can rise more vertically. This results in a gradual shift of its lateral location away from the older Hawaiian chain and toward the south (Figures 3E and 3F). Meanwhile, the eruption rate of the emerging plume head increases dramatically. Given that the Loa plume is

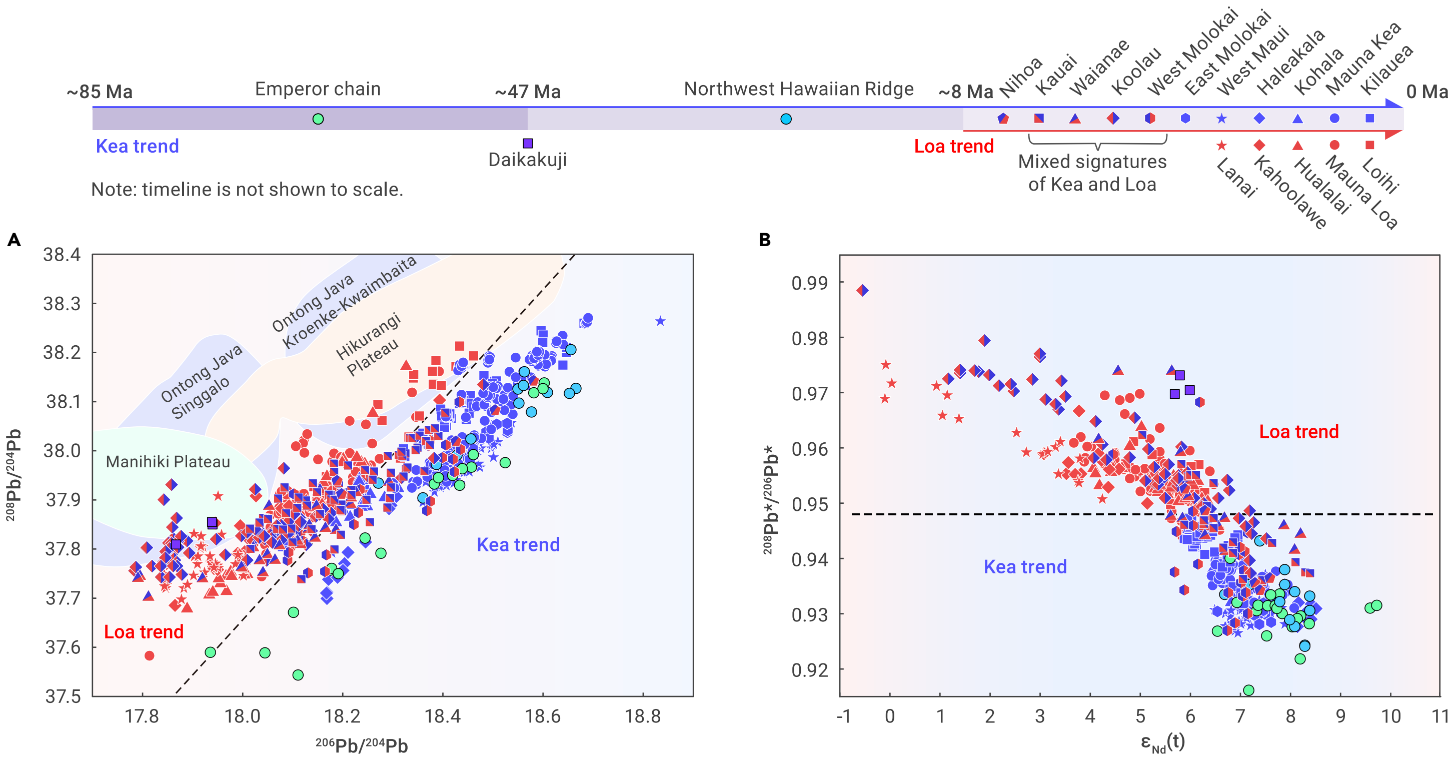

Figure 2. Isotope diagrams for shield-stage lavas of Hawaiian-Emperor volcano chains The Hawaiian-Emperor chain is divided into three sections: the Emperor chain, the Northwest Hawaiian Ridge, and the seamounts form Nihoa to the present position. (A) ^{208}Pb/^{204}Pb versus ^{206}Pb/^{204}Pb. The Hawaiian chain is divided into two distinctive trends, the Loa trend and the Kea trend, by the dashed line. Note that all samples from the Emperor chain and the Northwest Hawaiian Ridge except the Daikakuji seamount fall on the Kea trend, suggesting that the Kea trend was the original Hawaii chain, whereas the Loa trend is newly emerging. Nihoa, Kauai, Waianae, Koolau, and West Molokai have large ranges of isotopic compositions and fall on both trends, i.e., mixtures of materials from two trends at shallow depths. (B) A diagram of ^{208}Pb*/^{206}Pb* versus ε_{Nd}(t) where ^{208}Pb*/^{206}Pb* represents the time-integrated ^{232}Th/^{238}U ratio since the formation of the Earth and is defined as ^{208}Pb*/^{206}Pb* = (^{208}Pb/^{204}Pb−29.475)/(^{206}Pb/^{204}Pb−9.306).[28] Samples from the Loa trend all have ^{208}Pb*/^{206}Pb* higher than 0.948, with lower ε_{Nd}(t), representing an enriched mantle source. In contrast, samples from the Kea trend all have ^{208}Pb*/^{206}Pb* lower than 0.948, with higher ε_{Nd}(t), comparable to those of the depleted mantle. The sources of geochemical data of these seamounts are provided in the supplemental information.

broader and hotter than the Hawaiian plume tail conduit, the latter will eventually lean toward the former, whose more-negative dynamic pressure exerts a stronger suction force (Figures 3E and 3F). Finally, the two plume conduits stay close to, but remain separated from, each other, which could explain the two distinct compositional trends in the Hawaiian hotspot. Since ~5 Ma, the center of the plume system has experienced a continuous southward migration over ~175 km at 200 km depth (Figure 4), which is comparable to the southward migration of the surface hotspot (Figure 1).

This plume-plume interaction model we proposed is further supported by geophysical results. Global seismic tomography shows that there are two distinct anomalies underneath Hawaii.[54] The S-wave imaging also shows two low-velocity regions at depths of ~600 km. However, at depths of 900 and 1,200 km, the anomalous region to the northwest of the Hawaiian main island becomes vague, while that to the southeast is still clearly shown.[55] These results may imply that the Hawaiian plume tail is located to the northwest of the main island and is dying, whereas the young Loa plume is located to the southeast and is growing over time.

Implications on plume dynamics and resulting surface manifestation

According to the conventional plume hypothesis, the mantle source of plumes is largely stationary,[2,44] such that the island/seamount chain formed from volcanic eruptions within a moving plate. Paleomagnetic data showed that several Emperor seamounts older than 50 Ma erupted at paleolatitude far north of the current position, which has been interpreted due to southward migration of the plume in response to a change in the locus of upwelling mantle flow.[56–59] A recent study shows that this could be instead due to the interaction between the plume and the northward moving ridge between the Pacific and Izanagi plates.[60]

Here, we show that the surface expression of hotspots may also reflect a plume-plume interaction. Most previous models for Cenozoic variations in the Hawaiian chain involve a single mantle plume originating from either the ambient Pacific mantle or the large low shear-wave velocity province (LLSVP).[5,6,8,13,23–25] Of particular notice is the model by Harrison et al.,[13] who proposed that the once distant Hawaiian mantle plume sampling the ambient Pacific lowermost mantle moved toward and eventually interacted with the spatially fixed Pacific LLSVP, when the plume started to entrain the LLSVP material to form the Loa trend. This model satisfies multiple constraints but is inconsistent with geodynamic simulations showing that plumes tend to form above a temporally evolving LLSVP.[61–63] In addition, this model cannot explain the southward bending of the Hawaiian chain nor the recent increase in eruption rate.

In our model, both plumes could have originated from the top of the LLSVP. Their different isotopic signatures could be due to multiple reasons. One possibility is that their respective source regions have different compositions. A second possibility is that both plumes sample the same composition (i.e., the LLSVP), but their abilities to entrain the LLSVP material differ: the waning plume tail entrains less material than the newly formed plume head due to their different buoyancy and thus suction force applied to the underlying plume source. A third explanation is that both plumes entrain similar amounts of LLSVP material but the new plume head, due to its higher temperature and lack of prior melting, could express a stronger compositional signature.

Dynamically, the recent appearance of the Loa plume next to the older one may reflect a regional perturbation or reorganization of the convective regime within the lowermost mantle. According to recent geodynamic studies,[62,63] the LLSVP's configuration evolves over time due to push of the surrounding mantle and subducting slabs. We suggest that the formation of the new plume may be a result of this temporal variation. Furthermore, this recent lowermost mantle adjustment may also explain the two subparallel isotopic trends in Samoa and the Marquesas Islands that have similar ages to the Loa trend (Figure S1). We speculate that these hotspot tracks together reflect the same mechanism, i.e., an emerging new plume next to the old one, in response to a regional reorganization of the Pacific lower mantle. We propose that future work is needed to better understand the causes and consequences of this late-Cenozoic change in mantle dynamic regime.

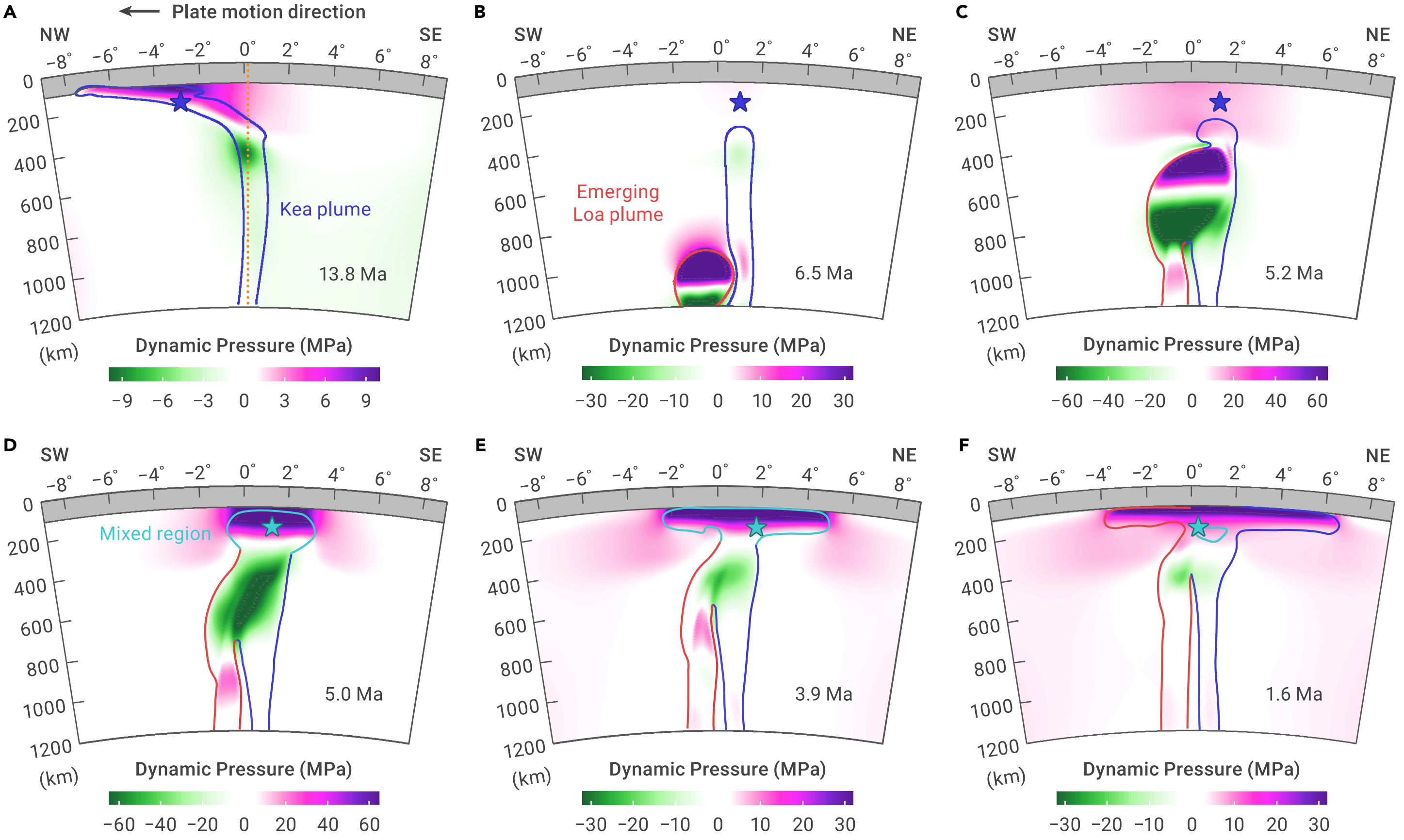

Figure 3. Temporal evolution of the double-plume system (A) The established Hawaiian plume at 13.8 Ma, prior to the commencement of the Loa plume. This cross-section is along the plate motion direction. The gray area and blue line represent the overlying oceanic lithosphere and the plume conduit, respectively. Within the plume conduit, dynamic pressure ranges from −9 to 6 MPa, with positive values right beneath the LAB and negative values in the deeper plume conduit. The dark blue stars denote spatial centers of the Kea plume system at 200 km depth. (B–F) Snapshots of the double-plume system at 6.5, 5.2, 5.0, 3.9, and 1.6 Ma, respectively. The light blue stars denote spatial centers of the double-plume system at 200 km depth. All cross-sections are perpendicular to the plate motion direction along the orange line in (A).

Mantle plume heads are often associated with large igneous provinces,[1,41,45] which can cause major global climate changes.[64–67] It has been proposed that the Deccan large igneous province in India was responsible for the mass extinction at the end of the Cretaceous.[64] It could also have contributed to the extremely hot climate in the early Cenozoic by adding CO_2 to the atmosphere. The eruption rate in Hawaii today is rapidly increasing, which we propose is due to invigoration from an emerging plume head. Considering the potentially major influences this increasing volcanic activity may have on the habitability of planet Earth, such a scenario is worthy of attention. However, if CO_2 in mantle plumes comes mainly from carbonated domains deep inside the mantle's transition zone,[68] the volatiles in the mantle domains underneath Hawaii may have already been undergoing extraction for more than 80 Myr. The emerging plume head would then likely be much more depleted in CO_2 and other volatile components compared with the Deccan plume head.

MATERIALS AND METHODS

Materials and methods related to this work are available in the supplemental information.

REFERENCES

1. Campbell, I.H. (2005). Large igneous provinces and the mantle plume hypothesis. Elements ***1***, 265–269.
2. Morgan, W.J. (1971). Convection plumes in lower mantle. Nature ***230***, 42–43.
3. Tatsumoto, M. (1978). Isotopic composition of lead in oceanic basalt and its implication to mantle evolution. Earth Planet Sci. Lett. ***38***, 63–87.
4. Hauri, E.H. (1996). Major-element variability in the Hawaiian mantle plume. Nature ***382***, 415–419.
5. Abouchami, W., Hofmann, A.W., Galer, S.J.G., et al. (2005). Lead isotopes reveal bilateral asymmetry and vertical continuity in the Hawaiian mantle plume. Nature ***434***, 851–856.
6. Huang, S., Hall, P.S., and Jackson, M.G. (2011). Geochemical zoning of volcanic chains associated with Pacific hotspots. Nat. Geosci. ***4***, 874–878.
7. Ren, Z.-Y., Ingle, S., Takahashi, E., et al. (2005). The chemical structure of the Hawaiian mantle plume. Nature ***436***, 837–840.
8. Weis, D., Garcia, M.O., Rhodes, J.M., et al. (2011). Role of the deep mantle in generating the compositional asymmetry of the Hawaiian mantle plume. Nat. Geosci. ***4***, 831–838.
9. Jackson, M.G., Weis, D., and Huang, S. (2012). Major element variations in Hawaiian shield lavas: source features and perspectives from global ocean island basalt (OIB) systematics. Geochem. Geophys. Geosyst. ***13***, 24.
10. Frey, F.A., Huang, S., Xu, G., and Jochum, K.P. (2016). The geochemical components that distinguish Loa- and Kea-trend Hawaiian shield lavas. Geochem. Cosmochim. Acta ***185***, 160–181.
11. Harrison, L.N., Weis, D., and Garcia, M.O. (2020). The multiple depleted mantle components in the Hawaiian-Emperor chain. Chem. Geol. ***532***, 119324.
12. Pietruszka, A.J., Norman, M.D., Garcia, M.O., et al. (2013). Chemical heterogeneity in the Hawaiian mantle plume from the alteration and dehydration of recycled oceanic crust. Earth Planet Sci. Lett. ***361***, 298–309.
13. Harrison, L.N., Weis, D., and Garcia, M.O. (2017). The link between Hawaiian mantle plume composition, magmatic flux, and deep mantle geodynamics. Earth Planet Sci. Lett. ***463***, 298–309.
14. Harrison, L.N., and Weis, D. (2018). The size and emergence of geochemical heterogeneities in the Hawaiian mantle plume constrained by Sr-Nd-Hf isotopic variation over similar to 47 million years. Geochem. Geophys. Geosyst. ***19***, 2823–2842.
15. Xu, G., Huang, S., Frey, F.A., et al. (2014). The distribution of geochemical heterogeneities in the source of Hawaiian shield lavas as revealed by a transect across the strike of the Loa and Kea spatial trends: east Molokai to West Molokai to Penguin Bank. Geochem. Cosmochim. Acta ***132***, 214–237.
16. Williamson, N.M.B., Weis, D., Scoates, J.S., et al. (2019). Tracking the geochemical transition between the kea-dominated northwest Hawaiian ridge and the bilateral loa-kea trends of the Hawaiian islands. Geochem. Geophys. Geosyst. ***20***, 4354–4369.
17. Sobolev, A.V., Hofmann, A.W., Sobolev, S.V., and Nikogosian, I.K. (2005). An olivine-free mantle source of Hawaiian shield basalts. Nature ***434***, 590–597.
18. Jones, T.D., Davies, D.R., Campbell, I.H., et al. (2017). The concurrent emergence and causes of double volcanic hotspot tracks on the Pacific plate. Nature ***545***, 472–476.
19. Hofmann, A.W., and Farnetani, C.G. (2013). Two views of Hawaiian plume structure. Geochem. Geophys. Geosyst. ***14***, 5308–5322.
20. Farnetani, C.G., and Hofmann, A.W. (2010). Dynamics and internal structure of the Hawaiian plume. Earth Planet Sci. Lett. ***295***, 231–240.

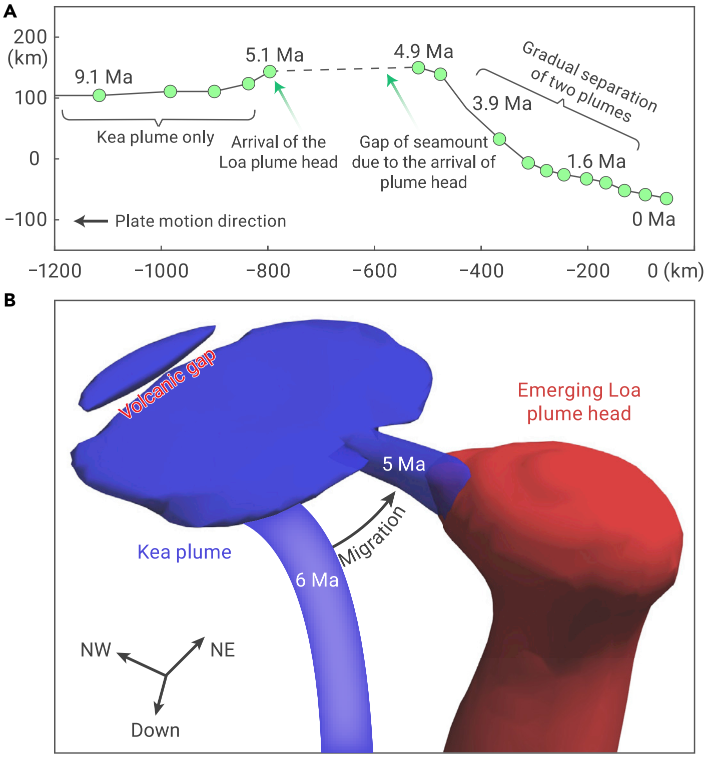

Figure 4. Predicted migration history of plume system centers and temporal evolution of the double-plume system in 3D view (A) Current locations of plume system centers identified at different time. The horizontal axis shows the distance to the current plume system center along the plate motion direction, where negative distance means that old centers are located west to the current center. The vertical axis shows the distance to current plume center in the direction perpendicular to the plate motion, where positive distance means that old centers are located north to the current center. Numbers next to the dots indicates the volcano ages. (B) The emerging Loa plume head causes the Kea plume conduit to migrate southeastward during 6–5 Ma, where the resulting ∼200 km volcanic gap is comparable to the observed surface magmatic hiatus.

21. Ballmer, M.D., Ito, G., van Hunen, J., and Tackley, P.J. (2011). Spatial and temporal variability in Hawaiian hotspot volcanism induced by small-scale convection. Nat. Geosci. ***4***, 457–460.
22. Xu, G., Frey, F.A., Clague, D.A., et al. (2007). Geochemical characteristics of West Molokai shield- and postshield-stage lavas: constraints on Hawaiian plume models. Geochem. Geophys. Geosyst. ***8***.
23. Lassiter, J.C., DePaolo, D.J., and Tatsumoto, M. (1996). Isotopic evolution of mauna Kea volcano: results from the initial phase of the Hawaii scientific drilling project. J. Geophys. Res. ***101***, 11769–11780.
24. Bryce, J.G., DePaolo, D.J., and Lassiter, J.C. (2005). Geochemical structure of the Hawaiian plume: Sr, Nd, and Os isotopes in the 2.8 km HSDP-2 section of Mauna Kea volcano. Geochem. Geophys. Geosyst. ***6***.
25. Huang, S., Abouchami, W., Blichert-Toft, J., et al. (2009). Ancient carbonate sedimentary signature in the Hawaiian plume: evidence from Mahukona volcano ***10***.
26. Wessel, P. (2016). Regional–residual separation of bathymetry and revised estimates of Hawaii plume flux. Geophys. J. Int. ***204***, 932–947.
27. Vidal, V., and Bonneville, A. (2004). Variations of the Hawaiian hot spot activity revealed by variations in the magma production rate. J. Geophys. Res. ***109***, B03104.
28. Galer, S.J.G., and Onions, R.K. (1985). Residence time of thorium, uranium and lead in the mantle with implications for mantle convection. Nature ***316***, 778–782.
29. Garcia, M.O., Swinnard, L., Weis, D., et al. (2010). Petrology, geochemistry and geochronology of Kaua'i lavas over 4·5 Myr: implications for the origin of rejuvenated volcanism and the evolution of the Hawaiian plume. J. Petrol. ***51***, 1507–1540.
30. Weis, D., Harrison, L.N., McMillan, R., and Williamson, N.M.B. (2020). Fine-Scale structure of earth's deep mantle resolved through statistical analysis of Hawaiian basalt geochemistry. Geochem. Geophys. Geosyst. ***21***, 18.
31. Tejada, M.L.G., Geldmacher, J., Hauff, F., et al. (2016). Geochemistry and age of shatsky, hess, and ojin rise seamounts: implications for a connection between the shatsky and hess rises. Geochem. Cosmochim. Acta ***185***, 302–327.
32. Tejada, M.L.G., Mahoney, J.J., Neal, C.R., et al. (2002). Basement geochemistry and geochronology of central malaita, Solomon Islands, with implications for the origin and evolution of the Ontong Java plateau. J. Petrol. ***43***, 449–484.
33. Tejada, M.L.G., Mahoney, J.J., Castillo, P.R., et al. (2004). Pin-pricking the elephant: evidence on the origin of the Ontong Java Plateau from Pb-Sr-Hf-Nd isotopic characteristics of ODP leg 192 basalts. Origin and Evolution of the Ontong Java Plateau ***229***, 133–150.
34. Mahoney, J.J., and Spencer, K.J. (1991). Isotopic evidence for the origin of the Manihiki and Ontong Java oceanic plateaus. Earth Planet Sci. Lett. ***104***, 196–210.
35. Ingle, S., Mahoney, J.J., Sato, H., et al. (2007). Depleted mantle wedge and sediment fingerprint in unusual basalts from the Manihiki Plateau, central Pacific Ocean. Geology ***35***, 595–598.
36. Hoernle, K., Hauff, F., van den Bogaard, P., et al. (2010). Age and geochemistry of volcanic rocks from the Hikurangi and Manihiki oceanic Plateaus. Geochem. Cosmochim. Acta ***74***, 7196–7219.
37. Timm, C., Hoernle, K., Werner, R., et al. (2011). Age and geochemistry of the oceanic Manihiki Plateau, SW Pacific: new evidence for a plume origin. Earth Planet Sci. Lett. ***304***, 135–146.
38. Garcia, M.O., Smith, J.R., Tree, J.P., et al. (2015). The Origin, Evolution, and Environmental Impact of Oceanic Large Igneous Provinces, ***511*** (Geological Society of America).
39. Tree, J.P. (2016). Mantle Potential Temperatures of 4.5 to 47 MA Hawaiian Volcanoes Using Olivine Thermometry: Implications for Melt Flux Variations (University of Hawaii at Manoa).
40. Campbell, I.H., and Griffiths, R.W. (1990). Implications of mantle plume structure for the evolution of flood basalts. Earth Planet Sci. Lett. ***99***, 79–93.
41. Coffin, M.F., and Eldholm, O. (1994). Large igneous provinces-crustal structure, dimensions, and external consequences. Rev. Geophys. ***32***, 1–36.
42. Koppers, A.A.P., Becker, T.W., Jackson, M.G., et al. (2021). Mantle plumes and their role in Earth processes. Nat. Rev. Earth Environ. ***2***, 382–401.
43. Whitehead, J.A., Jr., and Luther, D.S. (1975). Dynamics of laboratory diapir and plume models. J. Geophys. Res. ***80***, 705–717.
44. French, S.W., and Romanowicz, B. (2015). Broad plumes rooted at the base of the Earth'smantle beneath major hotspots. Nature ***525***, 95–99.
45. Richards, M.A., Duncan, R.A., and Courtillot, V.E. (1989). Flood basalts and hot-spot tracks: plume heads and tails. Science ***246***, 103–107.
46. Farnetani, C.G., and Samuel, H. (2005). Beyond the thermal plume paradigm. Geophys. Res. Lett. ***32***.
47. Wessel, P., and Müller, R.D. (2016). Ridge-spotting: a new test for Pacific absolute plate motion models. Geochem. Geophys. Geosyst. ***17***, 2408–2420.
48. Robinson, J.E., and Eakins, B.W. (2006). Calculated volumes of individual shield volcanoes at the young end of the Hawaiian Ridge. J. Volcanol. Geoth. Res. ***151***, 309–317.
49. Self, S., Widdowson, M., Thordarson, T., and Jay, A.E. (2006). Volatile fluxes during flood basalt eruptions and potential effects on the global environment: a Deccan perspective. Earth Planet Sci. Lett. ***248***, 518–532.
50. Bredow, E., Steinberger, B., Gassmöller, R., and Dannberg, J. (2017). How plume-ridge interaction shapes the crustal thickness pattern of the Réunion hotspot track. Geochem. Geophys. Geosyst. ***18***, 2930–2948.
51. Peters, B.J., and Day, J.M.D. (2017). A geochemical link between plume head and tail volcanism. Geochem. Perspect. Lett. ***5***, 29–34.
52. Griffiths, R.W., and Campbell, I.H. (1991). Interaction of mantle plume heads with the earths surface and onset of small-scale convection. J. Geophys. Res. ***96***, 18295–18310.
53. Zhong, S., McNamara, A., Tan, E., et al. (2008). A benchmark study on mantle convection in a 3-D spherical shell using CitcomS. Geochem. Geophys. Geosyst. ***9***.
54. Montelli, R., Nolet, G., Dahlen, F.A., et al. (2004). Finite-frequency tomography reveals a variety of plumes in the mantle. Science ***303***, 338–343.
55. Wolfe, C.J., Solomon, S.C., Laske, G., et al. (2009). Mantle shear-wave velocity structure beneath the Hawaiian hot spot. Science ***326***, 1388–1390.
56. Tarduno, J., Bunge, H.-P., Sleep, N., and Hansen, U. (2009). The bent Hawaiian-emperor hotspot track: inheriting the mantle wind. Science ***324***, 50–53.
57. Tarduno, J.A., Duncan, R.A., Scholl, D.W., et al. (2003). The emperor seamounts: southward motion of the Hawaiian hotspot plume in earth's mantle. Science ***301***, 1064–1069.
58. Torsvik, T.H., Doubrovine, P.V., Steinberger, B., et al. (2017). Pacific plate motion change caused the Hawaiian-Emperor Bend. Nat. Commun. ***8***, 15660.
59. Bono, R.K., Tarduno, J.A., and Bunge, H.-P. (2019). Hotspot motion caused the Hawaiian-Emperor Bend and LLSVPs are not fixed. Nat. Commun. ***10***, 3370.
60. Sun, W., Langmuir, C.H., Ribe, N.M., et al. (2021). Plume-ridge interaction induced migration of the Hawaiian-Emperor seamounts. Sci. Bull. ***66***, 1691–1697.
61. Burke, K., Steinberger, B., Torsvik, T.H., and Smethurst, M.A. (2008). Plume generation zones at the margins of large low shear velocity provinces on the core-mantle boundary. Earth Planet Sci. Lett. ***265***, 49–60.
62. Steinberger, B., and Torsvik, T.H. (2012). A geodynamic model of plumes from the margins of large low shear velocity provinces. Geochem. Geophys. Geosyst. ***13***.
63. Hassan, R., Müller, R.D., Gurnis, M., et al. (2016). A rapid burst in hotspot motion through the interaction of tectonics and deep mantle flow. Nature ***533***, 239–242.
64. Courtillot, V., and Olson, P. (2007). Mantle plumes link magnetic superchrons to phanerozoic mass depletion events. Earth Planet Sci. Lett. ***260***, 495–504.
65. Sobolev, S.V., Sobolev, A.V., Kuzmin, D.V., et al. (2011). Linking mantle plumes, large igneous provinces and environmental catastrophes. Nature ***477***, 312–316.
66. Self, S., Blake, S., Sharma, K., et al. (2008). Sulfur and chlorine in late Cretaceous Deccan magmas and eruptive gas release. Science ***319***, 1654–1657.
67. Hofmann, C., Courtillot, V., Féraud, G., et al. (1997). Timing of the Ethiopian flood basalt event and implications for plume birth and global change. Nature ***389***, 838–841.
68. Sun, W.-d., Hawkesworth, C.J., Yao, C., et al. (2018). Carbonated mantle domains at the base of the Earth's transition zone. Chem. Geol. ***478***, 69–75.

ACKNOWLEDGMENTS

This work was supported by the Strategic Priority Research Program of the Chinese Academy of Sciences (XDA22050103), the Marine S&T Fund of Shandong Province for Pilot

National Laboratory for Marine Science and Technology (Qingdao) (2022QNLM050201), and the National Natural Science Foundation of China (92258303) to W.S. L.L. acknowledges NSF grant EAR1554554.

AUTHOR CONTRIBUTIONS

W.S. and L.L. initiated this study and led the writing of the manuscript. L.Z. collected all the data and plotted figures. Z.C. carried out geodynamic modeling. All authors participated in discussion and writing.

DECLARATION OF INTERESTS

The authors declare no competing interests.

SUPPLEMENTAL INFORMATION

It can be found online at https://doi.org/10.1016/j.xinn.2023.100404.

LEAD CONTACT WEBSITE

http://ir.qdio.ac.cn/profile/weidongsun.

The Innovation, Volume *4*

Supplemental Information

An emerging plume head interacting with the Hawaiian plume tail

Lipeng Zhang, Zebin Cao, Robert E. Zartman, Congying Li, Saijun Sun, Lijun Liu, and Weidong Sun

1. Other double chains in the Pacific plate

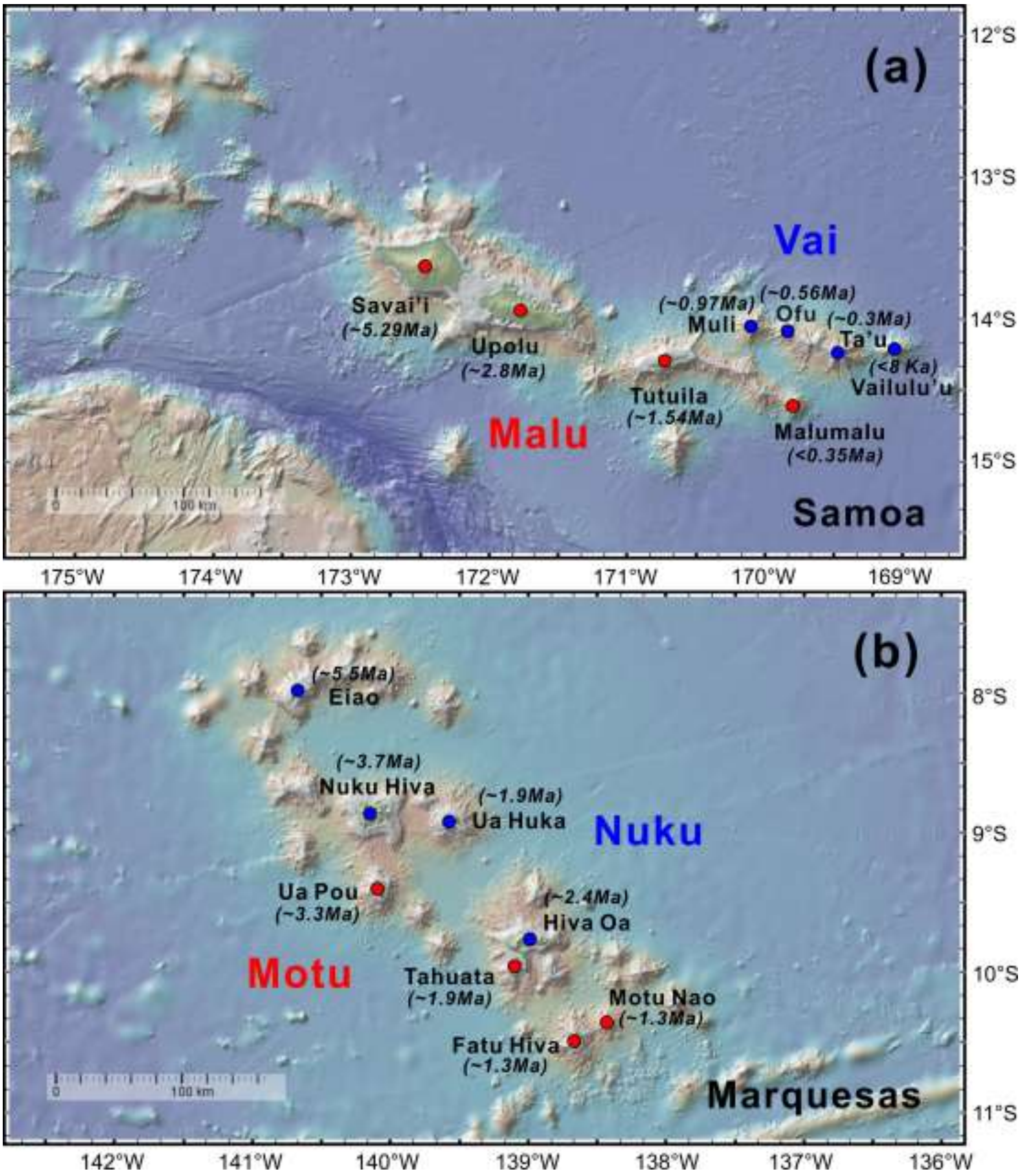

Figure S1 Map showing the other double volcano chains of **(a)** the Samoa and **(b)** the Marquesas plumes. In contrast to the Hawaiian plume, these two plumes are not bended nor increased in eruption. Also, these double chains appeared later than the Hawaiian double chain. The base maps are from the software of GeoMapApp. Age data are from literature as follow.

1. Jackson M, Hart S, Konter J, Kurz M, Blusztajn J, Farley K. Helium and lead isotopes reveal the geochemical geometry of the Samoan plume. *Nature* **514**, 355-

358 (2014).
2. Sims KW, et al. 238U-230Th-226Ra-210Pb-210Po, 232Th-228Ra, and 235U-231Pa constraints on the ages and petrogenesis of Vailulu'u and Malumalu Lavas, Samoa. *Geochemistry, Geophysics, Geosystems* **9**, (2008).
3. McDougall I. Age and evolution of the volcanoes of Tutuila, American Samoa. (1985).
4. Clouard V, Bonneville A. Ages of seamounts, islands, and plateaus on the Pacific plate. *Special Papers-Geological Society of America* **388**, 71 (2005).
5. Koppers AA, Russell JA, Jackson MG, Konter J, Staudigel H, Hart SR. Samoa reinstated as a primary hotspot trail. *Geology* **36**, 435-438 (2008).

2. Geochemical data for each island of the Hawaiian chain

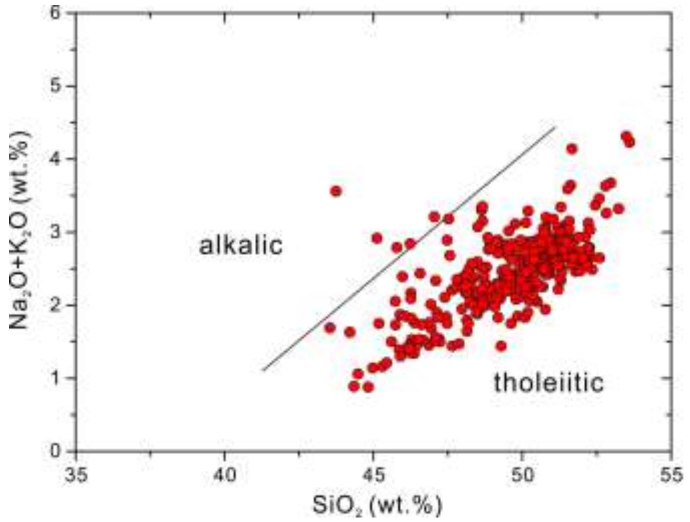

Figure S2 SiO_2 vs. total alkalis diagram. All the samples for which element concentrations are available were calculated as tholeiites. The tholeiitic/alkalic boundary is from Macdonald and Katsura (1964). The references used to determine the composition of each seamount are as follows.

Reference: Macdonald GA, Katsura T. Chemical Composition of Hawaiian Lavas1. *Journal of Petrology* **5**, (1964).

Data Sources for each island

Loihi

Abouchami, W., Hofmann, A.W., Galer, S.J., Frey, F.A., Eisele, J., Feigenson, M., 2005. Lead isotopes reveal bilateral asymmetry and vertical continuity in the Hawaiian mantle plume. Nature 434, 851.

Garcia, M.O., Foss, D.J.P., West, H.B., Mahoney, J.J., 1995. Geochemical and Isotopic Evolution of Loihi Volcano, Hawaii. Journal of Petrology 37, 1647-1674.

Garcia, M.O., Jorgenson, B.A., Mahoney, J.J., Ito, E., Irving, A.J., 1993. An evaluation of temporal geochemical evolution of Loihi Summit Lavas: Results from Alvin submersible dives. Journal of Geophysical Research Solid Earth 98, 537-550.

Mauna Loa

Abouchami, W., Galer, S.J.G., Hofmann, A.W., 2000. High precision lead isotope systematics of lavas from the Hawaiian Scientific Drilling Project. Chemical Geology 169, 187-209.

Blichert-Toft, J., Weis, D., Maerschalk, C., Agranier, A., Albarède, F., 2003. Hawaiian hot spot dynamics as inferred from the Hf and Pb isotope evolution of Mauna Kea volcano. Geochemistry Geophysics Geosystems 4.

Marske, J.P., Pietruszka, A.J., Weis, D., Garcia, M.O., Rhodes, J.M., 2007. Rapid passage of a small-scale mantle heterogeneity through the melting regions of Kilauea and Mauna Loa Volcanoes. Earth & Planetary Science Letters 259, 34-50.

Tanaka, R., Makishima, A., Nakamura, E., 2008. Hawaiian double volcanic chain triggered by an episodic involvement of recycled material: Constraints from temporal Sr–Nd–Hf–Pb isotopic trend of the Loa-type volcanoes. Earth & Planetary Science Letters 265, 450-465.

Tanaka, R., Nakamura, E., 2005. Boron isotopic constraints on the source of Hawaiian shield lavas. Geochimica et Cosmochimica Acta 69, 3385-3399.

Weis, D., Garcia, M.O., Rhodes, J.M., Jellinek, M., Scoates, J.S., 2011. Role of the deep mantle in generating the compositional asymmetry of the Hawaiian mantle plume. Nature Geoscience 4, 831-838.

Wanless, V.D., Garcia, M.O., Trusdell, F.A., Rhodes, J.M., Norman, M.D., Weis, D., Fornari, D.J., Kurz, M.D., Guillou, H., 2006. Submarine radial vents on Mauna Loa Volcano, Hawai'i. Geochemistry Geophysics Geosystems 7, 28.

Feigenson, M.D., Bolge, L.L., Carr, M.J., Herzberg, C.T., 2003. REE inverse modeling of HSDP2 basalts: Evidence for multiple sources in the Hawaiian plume. Geochemistry Geophysics Geosystems 4, 25.

Huailalai

Hanano, D., Weis, D., Scoates, J.S., Aciego, S., Depaolo, D.J., 2010. Horizontal and vertical zoning of heterogeneities in the Hawaiian mantle plume from the geochemistry of consecutive postshield volcano pairs: Kohala - Mahukona and Mauna Kea-Hualalai. Geochemistry Geophysics Geosystems 11.

Yamasaki, S., Kani, T., Hanan, B.B., Tagami, T., 2009. Isotopic geochemistry of Hualalai shield-stage tholeiitic basalts from submarine North Kona region, Hawaii. Journal of Volcanology & Geothermal Research 185, 223-230.

Kahoolawe

Abouchami, W., Hofmann, A.W., Galer, S.J., Frey, F.A., Eisele, J., Feigenson, M., 2005. Lead isotopes reveal bilateral asymmetry and vertical continuity in the Hawaiian mantle plume. Nature 434, 851.

Huang, S., Frey, F.A., Blichert-Toft, J., Fodor, R.V., Bauer, G.R., Xu, G., 2005. Enriched components in the Hawaiian plume: Evidence from Kahoolawe Volcano, Hawaii. Geochemistry Geophysics Geosystems 6.

Lanai

Abouchami, W., Hofmann, A.W., Galer, S.J., Frey, F.A., Eisele, J., Feigenson, M., 2005. Lead isotopes reveal bilateral asymmetry and vertical continuity in the Hawaiian mantle plume. Nature 434, 851.

Gaffney, A.M., Nelson, B.K., Blichert-Toft, J., 2005. Melting in the Hawaiian plume at 1-2 Ma as recorded at Maui Nui: The role of eclogite, peridotite, and source mixing. Geochemistry

Geophysics Geosystems 6.

Kilauea

Abouchami, W., Hofmann, A.W., Galer, S.J., Frey, F.A., Eisele, J., Feigenson, M., 2005. Lead isotopes reveal bilateral asymmetry and vertical continuity in the Hawaiian mantle plume. Nature 434, 851.

Marske, J.P., Pietruszka, A.J., Weis, D., Garcia, M.O., Rhodes, J.M., 2007. Rapid passage of a small-scale mantle heterogeneity through the melting regions of Kilauea and Mauna Loa Volcanoes. Earth & Planetary Science Letters 259, 34-50.

Tanaka, R., Makishima, A., Nakamura, E., 2008. Hawaiian double volcanic chain triggered by an episodic involvement of recycled material: Constraints from temporal Sr–Nd–Hf–Pb isotopic trend of the Loa-type volcanoes. Earth & Planetary Science Letters 265, 450-465.

Tanaka, R., Nakamura, E., 2005. Boron isotopic constraints on the source of Hawaiian shield lavas. Geochimica et Cosmochimica Acta 69, 3385-3399.

Hanyu T, Kimura J-I, Katakuse M, Calvert AT, Sisson TW, Naki Si., 2010. Source materials for inception stage Hawaiian magmas: Pb-He isotope variations for early Kilauea. Geochemistry Geophysics Geosystems 11.

Marske JP, Garcia MO, Pietruszka AJ, Rhodes JM, Norman MD., 2008. Geochemical variations during Kilaueas Pu'u eruption reveal a fine-scale mixture of mantle heterogeneities within the Hawaiian plume. Journal of Petrology 49, 1297-1318.

Pietruszka AJ, Norman MD, Garcia MO, Marske JP, Burns DH., 2013. Chemical heterogeneity in the Hawaiian mantle plume from the alteration and dehydration of recycled oceanic crust. Earth and Planetary Science Letters 361, 298-309.

Mauna Kea

Abouchami, W., Galer, S.J.G., Hofmann, A.W., 2000. High precision lead isotope systematics of lavas from the Hawaiian Scientific Drilling Project. Chemical Geology 169, 187-209.

Abouchami, W., Hofmann, A.W., Galer, S.J., Frey, F.A., Eisele, J., Feigenson, M., 2005. Lead isotopes reveal bilateral asymmetry and vertical continuity in the Hawaiian mantle plume. Nature 434, 851.

Blichert-Toft, J., Weis, D., Maerschalk, C., Agranier, A., Albarède, F., 2003. Hawaiian hot spot dynamics as inferred from the Hf and Pb isotope evolution of Mauna Kea volcano. Geochemistry Geophysics Geosystems 4.

Eisele, J., Abouchami, W., Galer, S.J.G., Hofmann, A.W., 2003. The 320 kyr Pb isotope evolution of Mauna Kea lavas recorded in the HSDP‐2 drill core. Geochemistry Geophysics Geosystems 4.

Hanano, D., Weis, D., Scoates, J.S., Aciego, S., Depaolo, D.J., 2010. Horizontal and vertical zoning of heterogeneities in the Hawaiian mantle plume from the geochemistry of consecutive postshield volcano pairs: Kohala‐Mahukona and Mauna Kea-Hualalai. Geochemistry Geophysics Geosystems 11.

Rhodes, J.M., Vollinger, M.J., 2004. Composition of basaltic lavas sampled by phase‐2 of the Hawaii Scientific Drilling Project: Geochemical stratigraphy and magma types. Geochemistry Geophysics Geosystems 5, -.

Silva, I.G.N., Weis, D., Scoates, J.S., 2013. Isotopic systematics of the early Mauna Kea shield phase and insight into the deep mantle beneath the Pacific Ocean. Geochemistry Geophysics Geosystems 14, 659-676.

Huang, S., Frey, F.A., 2003. Trace element abundances of Mauna Kea basalt from phase 2 of the Hawaii Scientific Drilling Project: Petrogenetic implications of correlations with major element content and isotopic ratios. Geochemistry Geophysics Geosystems 4, 43.

Kohala

Abouchami, W., Hofmann, A.W., Galer, S.J., Frey, F.A., Eisele, J., Feigenson, M., 2005. Lead isotopes reveal bilateral asymmetry and vertical continuity in the Hawaiian mantle plume. Nature 434, 851.

Hanano, D., Weis, D., Scoates, J.S., Aciego, S., Depaolo, D.J., 2010. Horizontal and vertical zoning of heterogeneities in the Hawaiian mantle plume from the geochemistry of consecutive postshield volcano pairs: Kohala - Mahukona and Mauna Kea-Hualalai. Geochemistry Geophysics Geosystems 11,

Haleakala

Phillips, E.H., Sims, K.W.W., Sherrod, D.R., Salters, V.J.M., Blusztajn, J., Dulai, H., 2016. Isotopic constraints on the genesis and evolution of basanitic lavas at Haleakala, Island of Maui, Hawaii. Geochimica et Cosmochimica Acta 195, 201-225.

West Maui

Gaffney, A.M., Nelson, B.K., Blicherttoft, J., 2004. Geochemical Constraints on the Role of Oceanic Lithosphere in Intra-Volcano Heterogeneity at West Maui, Hawaii. Journal of Petrology 45, 1663-1687(1625).

East Molokai

Xu, G., Frey, F.A., Clague, D.A., Weis, D., Beeson, M.H., 2005. East Molokai and other Kea - trend volcanoes: Magmatic processes and sources as they migrate away from the Hawaiian hot spot. Geochemistry Geophysics Geosystems 6.

West Molokai

Xu, G., Frey, F.A., Clague, D.A., Abouchami, W., Blichert-Toft, J., Cousens, B., Weisler, M., 2007. Geochemical characteristics of West Molokai shield- and postshield-stage lavas: Constraints on Hawaiian plume models. Geochemistry Geophysics Geosystems 8.

Koolau

Abouchami, W., Hofmann, A.W., Galer, S.J., Frey, F.A., Eisele, J., Feigenson, M., 2005. Lead isotopes reveal bilateral asymmetry and vertical continuity in the Hawaiian mantle plume. Nature 434, 851.

Fekiacova, Z., Abouchami, W., Galer, S.J.G., Garcia, M.O., Hofmann, A.W., 2007. Origin and temporal evolution of Koʻolau Volcano, Hawaiʻi: Inferences from isotope data on the Koʻolau Scientific Drilling Project (KSDP), the Honolulu Volcanics and ODP Site 843. Earth & Planetary Science Letters 261, 65-83.

Roden, M.F., Trull, T., Hart, S.R., Frey, F.A., 1994. New He, Nd, Pb, and Sr isotopic constraints on the constitution of the Hawaiian plume: Results from Koolau Volcano, Oahu, Hawaii, USA. Geochimica et Cosmochimica Acta 58, 1431-1440.

Salters, V.J.M., Blichert-Toft, J., Fekiacova, Z., Sachi-Kocher, A., Bizimis, M., 2006. Isotope and trace element evidence for depleted lithosphere in the source of enriched Ko'olau basalts. Contributions to Mineralogy & Petrology 151, 297-312.

Tanaka, R., Makishima, A., Nakamura, E., 2008. Hawaiian double volcanic chain triggered by an episodic involvement of recycled material: Constraints from temporal Sr–Nd–Hf–Pb isotopic trend of the Loa-type volcanoes. Earth & Planetary Science Letters 265, 450-465.

Tanaka, R., Nakamura, E., Takahashi, E., 2002. Geochemical evolution of Koolau Volcano, Hawaii. Hawaiian Volcanoes: Deep Underwater Perspectives 128, 311-332.

Haskins EH, Garcia MO., 2004. Scientific drilling reveals geochemical heterogeneity within the Ko'olau shield, Hawai'i. Contributions to Mineralogy and Petrology 147, 162-188.

Huang S, Frey FA., 2005. Recycled oceanic crust in the Hawaiian Plume: evidence from temporal geochemical variations within the Koolau Shield. Contributions to Mineralogy & Petrology 149, 556-575.

Waianae

Coombs, M., Clague, D.A., Moore, J.G., Cousens, B.L., 2004. Growth and collapse of Waianae Volcano, Hawaii, as revealed by exploration of its submarine flanks. Geochem. Geophys. Geosyst. 5, Q08006.

Van der Zander, I., Sinton, J.M., Mahoney, J.J., 2010. Late shield-stage silicic magmatism at Wai‘anae volcano: Evidence for hydrous crustal melting in Hawaiian volcanoes. Journal of Petrology 51, 671-701.

Williamson, N.M.B., Weis, D., Scoates, J.S., Pelletier, H., Garcia, M.O., 2019. Tracking the Geochemical Transition Between the Kea-Dominated Northwest Hawaiian Ridge and the Bilateral Loa-Kea Trends of the Hawaiian Islands. Geochemistry Geophysics Geosystems 20, 4354-4369.

Kauai

Garcia, M.O., Swinnard, L., Weis, D., Greene, A.R., Tagami, T., Sano, H., Gandy, C.E., 2010. Petrology, Geochemistry and Geochronology of Kaua‘i Lavas over 4.5 Myr: Implications for the Origin of Rejuvenated Volcanism and the Evolution of the Hawaiian Plume. Journal of Petrology 51, 1507-1540.

Mukhopadhyay, S., Lassiter, J.C., Farley, K.A., Bogue, S.W., 2003. Geochemistry of Kauai shield-stage lavas: Implications for the chemical evolution of the Hawaiian plume. Geochemistry Geophysics Geosystems 4, 32.

Williamson, N.M.B., Weis, D., Scoates, J.S., Pelletier, H., Garcia, M.O., 2019. Tracking the Geochemical Transition Between the Kea-Dominated Northwest Hawaiian Ridge and the Bilateral Loa-Kea Trends of the Hawaiian Islands. Geochemistry Geophysics Geosystems 20, 4354-4369.

Nihoa

Harrison, L.N., Weis, D., Garcia, M.O., 2017. The link between Hawaiian mantle plume composition, magmatic flux, and deep mantle geodynamics. Earth and Planetary Science Letters 463, 298-309.

Harrison, L.N., Weis, D., 2018. The Size and Emergence of Geochemical Heterogeneities in the Hawaiian Mantle Plume Constrained by Sr-Nd-Hf Isotopic Variation Over similar to 47 Million Years. Geochemistry Geophysics Geosystems 19, 2823-2842.

Northwest Hawaiian Rigde

Harrison, L.N., Weis, D., 2018. The Size and Emergence of Geochemical Heterogeneities in the Hawaiian Mantle Plume Constrained by Sr-Nd-Hf Isotopic Variation Over similar to 47 Million Years. Geochemistry Geophysics Geosystems 19, 2823-2842.

Harrison, L.N., Weis, D., Garcia, M.O., 2017. The link between Hawaiian mantle plume composition, magmatic flux, and deep mantle geodynamics. Earth and Planetary Science Letters 463, 298-309.

Emperor Chain

Regelous, M., Hofmann, A.W., Abouchami, W., Galer, S.J.G., 2003. Geochemistry of lavas from the Emperor Seamounts, and the geochemical evolution of Hawaiian magmatism from 85 to 42 Ma. Journal of Petrology 44, 113-140(128).

Keller, R.A., Fisk, M.R., White, W.M., 2000. Isotopic evidence for Late Cretaceous plume-ridge interaction at the Hawaiian hotspot. Nature 405, 673-676.

Garcia, M.O., Smith, J.R., Tree, J.P., Weis, D., Harrison, L., Jicha, B.R., 2015. "Petrology, geochemistry, and ages of lavas from Northwest Hawaiian Ridge volcanoes", The Origin, Evolution, and Environmental Impact of Oceanic Large Igneous Provinces, Clive R. Neal, William W. Sager, Takashi Sano, Elisabetta Erba

Tree, J., 2016. Mantle Potential Temperatures of 4.5 to 47 Ma Hawaiian Volcanoes Using Olivine Thermometry: Implications for Melt Flux Variations. A master's thesis of the University of Hawaiʻi at Mānoa

Ontong Java

Tejada, M.L.G., Mahoney, J.J., Castillo, P.R., Ingle, S.P., Sheth, H.C., Weis, D., 2004. Pin-pricking the elephant: evidence on the origin of the Ontong Java Plateau from Pb-Sr-Hf-Nd isotopic characteristics of ODP leg 192 basalts. Origin and Evolution of the Ontong Java Plateau 229, 133-150.

Tejada, M.L.G., Mahoney, J.J., Neal, C.R., Duncan, R.A., Petterson, M.G., 2002. Basement geochemistry and geochronology of Central Malaita, Solomon Islands, with implications for the origin and evolution of the Ontong Java Plateau. Journal of Petrology 43, 449-484.

Tejada, M.L.G., Suzuki, K., Hanyu, T., Mahoney, J.J., Ishikawa, A., Tatsumi, Y., Chang, Q., Nakai, S., 2013. Cryptic lower crustal signature in the source of the Ontong Java Plateau revealed by Os and Hf isotopes. Earth and Planetary Science Letters 377-378, 84-96.

Manihiki Plateau

Hoernle, K., Hauff, F., van den Bogaard, P., Werner, R., Mortimer, N., Geldmacher, J., Garbe-Schönberg, D., Davy, B., 2010. Age and geochemistry of volcanic rocks from the Hikurangi and Manihiki oceanic Plateaus. Geochimica Et Cosmochimica Acta 74, 7196-7219.

Ingle, S., Mahoney, J.J., Sato, H., Coffin, M., Kimura, J.I., Hirano, N., Nakanishi, M., 2007. Depleted mantle wedge and sediment fingerprint in unusual basalts from the Manihiki Plateau, central Pacific Ocean. Geology 35, 595-598.

Mahoney, J.J., Spencer, K.J., 1991. Isotopic evidence for the origin of the Manihiki and Ontong Java oceanic plateaus. Earth and Planetary Science Letters 104, 196-210.

Timm, C., Hoernle, K., Werner, R., Hauff, F., den Bogaard, P.v., Michael, P., Coffin, M.F., Koppers, A., 2011. Age and geochemistry of the oceanic Manihiki Plateau, SW Pacific: New evidence for a plume origin. Earth and Planetary Science Letters 304, 135-146.

Hikurangi Plateau
Hoernle, K., Hauff, F., van den Bogaard, P., Werner, R., Mortimer, N., Geldmacher, J., Garbe-Schönberg, D., Davy, B., 2010. Age and geochemistry of volcanic rocks from the Hikurangi and Manihiki oceanic Plateaus. Geochimica Et Cosmochimica Acta 74, 7196-7219.

3. Geodynamic model setup

To study plume-plume interaction, we used a user-updated version of 3D spherical finite element code CitcomS. On finite-element mesh, we solved the conservation equations of mass, momentum, and energy, under the Boussinesq approximation:

$$\nabla \cdot \vec{u} = 0$$

$$-\nabla P + \nabla \cdot [\eta(\nabla \vec{u} + \nabla^T \vec{u})] + \rho_m \alpha \Delta T \vec{g} = 0$$

$$\frac{\partial T}{\partial t} + \vec{u} \cdot \nabla T = \kappa \nabla^2 T$$

where $\vec{u}$ is velocity, P is dynamic pressure, η is effective viscosity, ρ_m is reference mantle density, α is thermal expansion coefficient, ΔT is thermal anomaly, $\vec{g}$ is gravitational acceleration, T is temperature, and κ is thermal diffusivity, respectively.

The model spans 30° × 30° × 2000 km in longitude × latitude × radius, a region much larger than the Hawaiian volcanic chain. The vertical and horizontal resolution are 15 km and 6 km, respectively. Passive tracers are used to track the evolution of two plumes, assuming no extra chemical buoyancy of plume material.

Boundary conditions involve temperature and velocity boundary conditions at surface. The surface potential temperature is set to be 0 °C, beneath the surface we set up a thermal oceanic lithosphere based on half-space cooling model, assuming 80 Myr old seafloor. The surface has been prescribed a constant westward plate motion at 10 cm/yr through the whole simulation.

To model realistic plume structure above 1000 km depth, we fixed heat sources as cones at depth from 1400 km to 1500 km. The resultant plume conduits have spatial averaged excess temperatures varying from 100 °C to 300 °C at LAB depth (~100 km). The present plume conduit under Hawaii has a spatial averaged excess temperature around 300 °C, which is consistent with literature estimates[5,6]. The heat source for Loa trend is located 220 km south to the one for Kea trend, and its base radius is twice as large as that for Kea trend. To simulate the evolution of Hawaiian volcanic chain, we started with the heat source for Kea trend only. After 40 Myr, the Kea trend is formed and became steady. At this time, the heat source for Loa trend was added (Figure S3).

Temperature- and depth-dependent Newtonian rheology is applied for the whole model domain. The background mantle viscosity at the ambient mantle temperature has a 3 layer-profile: lithosphere (0-100km), asthenosphere (100-410 km), and below (410-2000 km). Their respective

viscosity values are 0.1*η_0 (10^{20} Pa s), 0.1*η_0 (10^{20} Pa s), η_0 (10^{21} Pa s), where η_0 is the reference viscosity (10^{21} Pa s). The temperature-dependent Newtonian rheology follows:

$$\eta = \eta_{b.g.}(\mathrm{r})\exp(\frac{E(r)}{T+T_0} - \frac{E(r)}{T_m+T_0})$$

where η is effective viscosity, $\eta_{b.g.}$ is background mantle viscosity, E is activation energy, T is temperature, T_0 is temperature offset, and T_m is ambient mantle temperature. The activation energy used in this study is the same as in Hu et al. (2018). With strong temperature-dependent Newtonian rheology, our strong lithosphere reaches 10^{23} Pa s in the upper part and smoothly transient to weak asthenosphere. Other physical parameters used are shown in table S2.

References:

1. Li, X., Kind, R., Priestley, K., Sobolev, S. V., Tilmann, F., Yuan, X., & Weber, M. (2000). Mapping the Hawaiian plume conduit with converted seismic waves. Nature, 405(6789), 938-941.
2. Putirka, K. (2008). Excess temperatures at ocean islands: Implications for mantle layering and convection. Geology, 36(4), 283-286.
3. Hu, J., Liu, L., & Zhou, Q. (2018). Reproducing past subduction and mantle flow using high - resolution global convection models. Earth and Planetary Physics, 2(3), 189-207.

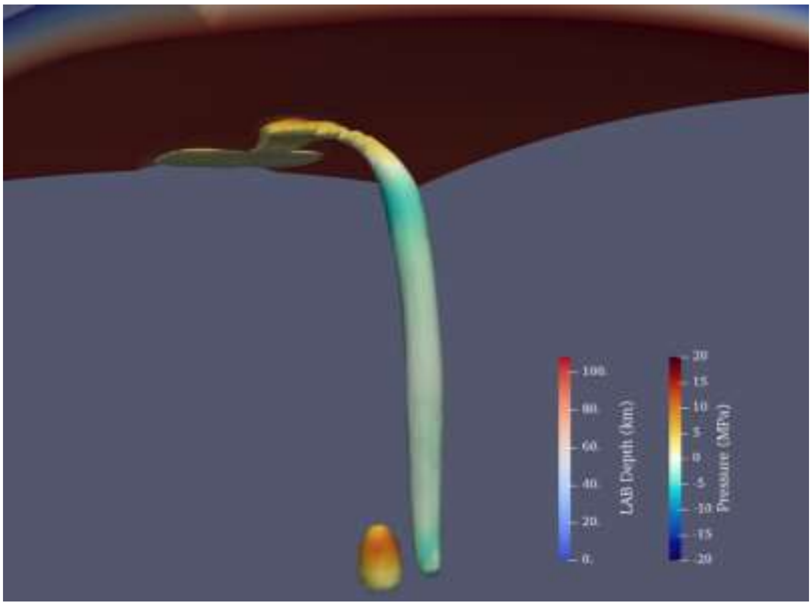

Figure S3 3D view of the Hawaiian plume system at 40 Myr from starting time, when the Loa plume emerged from the lower mantle. View towards northwest direction. The right plume is established Kea plume, the left one is newly formed Loa plume which has a larger size than the Kea plume. Plumes are presented by isothermal surface with 50 ˚C excess temperature.

Table S2 Physical parameters used in geodynamic model

Parameter	Symbol	Value	Unit
Earth radius	R	6371.0	km
Gravitational acceleration	g	9.81	m/s^2
Reference mantle density	ρ	3340	kg/m^3
Reference viscosity	η_0	10^{21}	Pa s
Background viscosity	$\eta_{b.k.}$	10^{20}, 10^{20}, 10^{21}	Pa s
Thermal diffusivity	κ	10^{-6}	m^2/s
Thermal expansivity	α	3.0×10^{-5}	1/°C
Rayleigh number	Ra	5.0e8	-
Activation energy	E	100, 166, 100	kJ/mol
Minimum viscosity	η_{min}	1.0e19	Pa s
Maximum viscosity	η_{max}	1.0e23	Pa s

4. Migration history of plume system center

We identified the centers of Hawaiian plume system in our geodynamic model at 200 km depth which can be used to show the migration of surface volcanic activities. The hottest part of the Hawaiian plume system is defined as the center of the plume system at each output timestep. Since melting processes highly depend on the P-T condition, the hottest part may well represent the center of the plume system with the highest melt production rate at this depth. After identification, the centers were assumed to move with the oceanic lithosphere at the same speed. The temporal and spatial evolution of plume system center is shown in Figure 5 in the main text.
